# Intracellular Fe^2+^ accumulation in endothelial cells and pericytes induces blood-brain barrier dysfunction in secondary brain injury after brain hemorrhage

**DOI:** 10.1038/s41598-019-42370-z

**Published:** 2019-04-17

**Authors:** Takahiko Imai, Sena Iwata, Tasuku Hirayama, Hideko Nagasawa, Shinsuke Nakamura, Masamitsu Shimazawa, Hideaki Hara

**Affiliations:** 1 Molecular Pharmacology, Department of Biofunctional Evaluation, Gifu Pharmaceutical University, Gifu, 501-1196 Japan; 2 Pharmaceutical and Medicinal Chemistry, Gifu Pharmaceutical University, Gifu, 501-1196 Japan

**Keywords:** Apoptosis, Blood-brain barrier, Cell death in the nervous system, Cell death in the nervous system, Stroke

## Abstract

After intracranial hemorrhage (ICH), iron is released from the hematoma and induces secondary brain injury. However, the detail effect of iron on blood-brain barrier (BBB) function is still unknown. We investigated whether hemoglobin (Hb), ferrous ammonium sulfate (FAS) or hemin which contains iron have the detrimental effect on both human brain microvascular endothelial cells and pericytes by cellular function analysis *in vitro*. We developed an iron (Fe^2+^)-detectable probe, Si-RhoNox-1, to investigate intracellular Fe^2+^ accumulation (Fe^2+^_intra_). After FAS treatment, there was the correlation between Fe^2+^_intra_ and cell death. Moreover, Hb or hemin treatment induced cell death, increased reactive oxygen species and promoted Fe^2+^_intra_ in both cells. These changes were inhibited by the Fe^2+^ chelator, 2,2′-bipyridil (BP). Furthermore, hemin induced endothelial barrier dysfunction via disruption of junction integrity. Based on *in vitro* studies, we used a hemin-injection ICH mice model *in vivo*. Hemin injection (10 mM/10 µL, i.c.) induced deleterious effects including BBB hyper-permeability, neuronal deficits, neuronal damage, altered proteins expression, and Fe^2+^_intra_ in BBB composed cells. Lastly, BP (40 mg/kg, i.p.) administration attenuated neuronal deficits at 3 days after surgery. Collectively, Hb or hemin damaged BBB composed cells via Fe^2+^_intra_. Therefore, the regulation of the Fe^2+^ movement in BBB might be effective for treatment of ICH.

## Introduction

Intracranial hemorrhage (ICH) is a devastating disease which causes high mortality and morbidity, accounting 20% of all stroke cases^1–3^. After hemorrhage, the hematoma formation and expansion induce directly brain parenchyma damage through mechanical effect at an early stage, and this damage is defined as “primary brain injury”^1,4^. Contrary to that, hematoma components induce “secondary brain injury” that is exemplified by neuronal cell death, inflammation, and brain edema formation at later stage^2,3,5,6^. To improve patients outcome, management of intracerebral pressure and blood pressure are important in ICH therapeutic guideline. However, these therapeutic approaches are symptomatic treatment, and there are no effective drugs for neuronal deficit at later phase after ICH^7,8^. In addition, although there are many basic researches that several neuroprotective drugs improved on ICH animal models, no one has been approved in clinical stage.

Previous studies utilizing autologous blood injection ICH model suggest that extravasated blood plays a deteriorative role via several pathways (apoptosis, autophagy, and ferroptosis etc.) and several stresses (oxidative and inflammatory stresses etc.)^1,4,9–12^. Following hematoma formation, Hb which is contained in red blood cells is released from the hematoma into the brain parenchyma tissue within hours to days. Leaked Hb and heme (or hemin) are gradually released into surrounding normal tissue and subsequently metabolized to carbon monoxide, biliverdin and iron by heme oxygenase-1 or -2 (HO-1/2)^13^. *In vivo* and *in vitro* models have shown that Hb and heme (hemin) have cytotoxic effects on neurons via inflammatory reaction^1,5,12–18^. Therefore, Hb and hemin are key mediators in neuronal damage, and ultimately induce secondary brain injury after ICH.

In the animal ICH model, brain iron accumulation in the perihematoma region gradually increased at day 3 after ICH and peaked at day 14, and the iron was detected in neurons, microglia, astrocytes and endothelial cells at day 14 after ICH^17^. Extracellular and intracellular iron accumulation accelerates reactive oxygen species (ROS) production and cellular lipid peroxidation by the Fenton reaction (Fe^2+^ + H_2_O_2_ → Fe^3+^ + HO^−^ + HO•)^19,20^. In fact, many previous studies have indicated that there was the relationship between iron accumulation and poor outcome after ICH^6,21–23^. Based on the correlation between both iron accumulation and ICH damage, several studies have suggested that Hb/heme scavenger proteins (e.g. hemopexin and haptoglobin) and iron chelators (e.g. deferoxamine) may be useful for the prevention of secondary brain injury after ICH in the clinical phase^22,24–26^. However, the protective effect on BBB has been controversial yet.

Endothelial cells and pericytes play important roles in both BBB maintenance and regulation of cell-to-cell interactions with astrocytes, microglia and neurons^27,28^. In the hemorrhagic condition, BBB integrity is disrupted by a decrease in endothelial cell-cell junction proteins and the dissociation of pericytes from the endothelium membrane^4,29,30^. Previous studies utilizing experimental stroke models have shown that BBB compromise accelerates blood leakage, which results in brain edema^1,12,16^. Moreover, our previous reports utilizing an experimental stroke model suggested that preserving endothelial cells and pericytes viability improved poor outcome of brain hemorrhagic events such as collagenase-induced ICH and hemorrhage transformation^29,30^. However, the detailed mechanism of Hb or hemin-mediated effects on BBB composed cells in hemorrhagic conditions is not clear. Particularly, the role of intracellular iron is unknown. Therefore, elucidating the mechanism of Hb or hemin-mediated BBB damage via iron accumulation may be useful for the development of a novel therapeutic strategy for the treatment of secondary brain injury after ICH.

In the present study, we hypothesized that leaked Hb/heme damages BBB after ICH and which leads to secondary brain injury. Therefore, we utilized an *in vitro* cell damage model and *in vivo* hemin injection model to investigate that Hb or hemin has the harmful effects on BBB composed cells such as endothelial cells and pericytes. To our knowledge, this is the first report demonstrating that non-heme or heme-binding iron accumulates in human brain microvascular cells (endothelial cells and pericytes) and induces cell death via increasing ROS production. This report also documents the novel finding that hemin injures BBB composed cells and BP has a protective effect on secondary brain injury after hemin injection.

## Results

All experimental detailed data are described in Supplemental materials.

### Human Hb damaged BBB composed cells via inducing ROS over-production and BP ameliorated Hb-induced harmful effects

**Figure 1:**
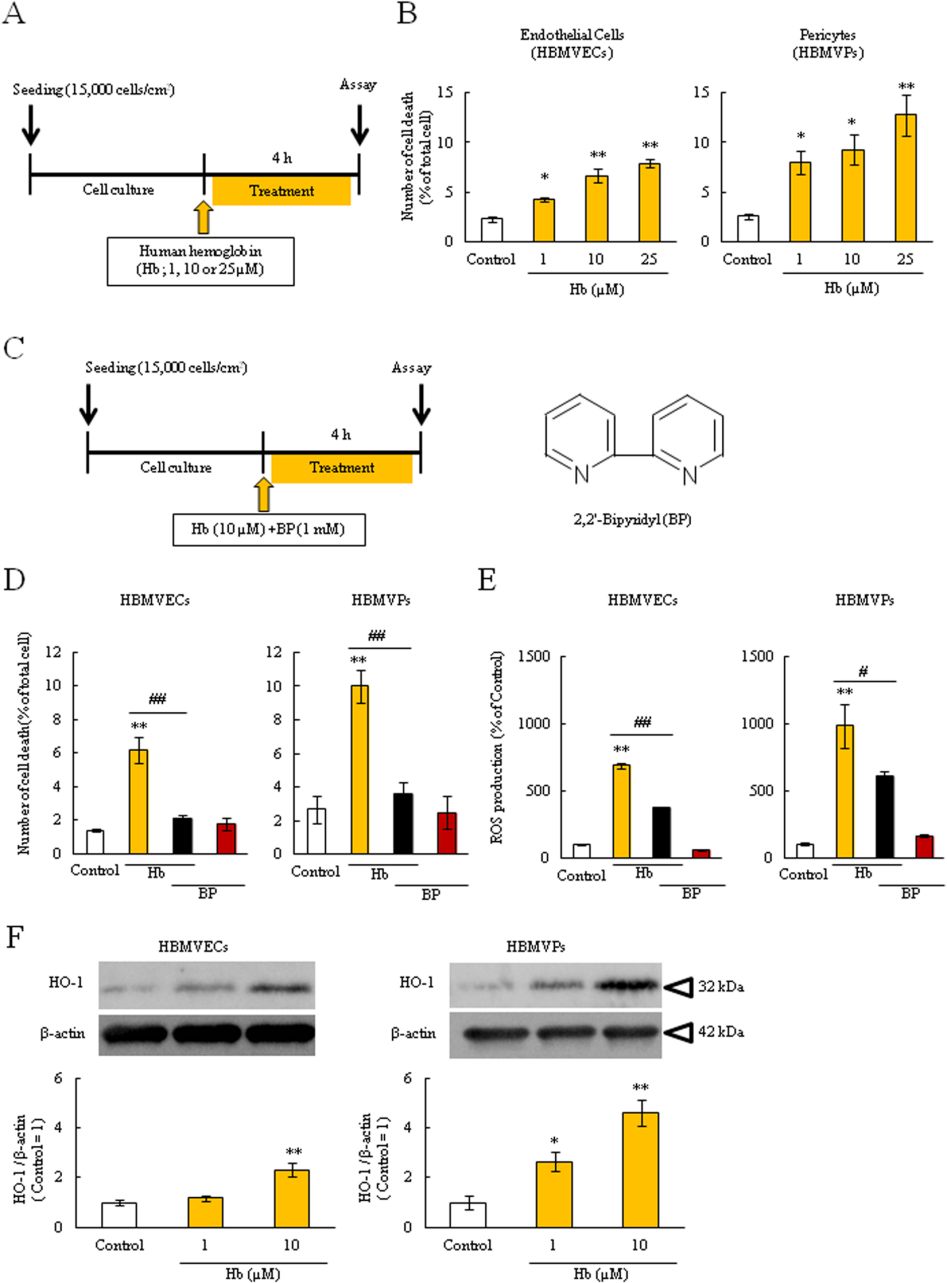
Hb induced cell death and ROS over-production in endothelial cells and pericytes. (**A**) Experimental protocol of the cell death assay after human hemoglobin (Hb) treatment (1, 10 or 25 µM). (**B**) Human brain microvascular endothelial cells (HBMVECs) and pericytes (HBMVPs) were incubated with Hb for 4 hours. The number of PI and Hoechst 33342-positive cells was counted, and the cell death rate was calculated as a percentage of PI-positive to Hoechst 33342-positive cells (n = 4). (**C**) Experimental protocol of the cell death and ROS assay, and the structural formula of 2,2′-bipyridil (BP). BP is a lipid-soluble Fe^2+^ chelator. (**D**) Cells were incubated with Hb (10 µM) and BP (1 mM) for 4 hours. The cell death rate is shown (n = 6). (**E**) The ROS production rate was corrected by the number of living cells (n = 6). (**F**) The expression of heme oxygenase-1 (HO-1). The upper images are representative bands and the lower graphs comprise the quantitative data (n = 4). (**D**) **p < 0.01, *p < 0.05 vs. Control; ^##^p < 0.01, ^#^p < 0.05 vs. Hb. The data was analyzed with the Dunnett’s test (**B**,**F**) or the Tukey’s test (**D**,**E**). The data are expressed as the mean ± SE.

To evaluate the effects of Hb on BBB composed cells, we assessed the cell death rate of both cells after Hb treatment for 4 h by using *in vitro* monoculture model such as endothelial cells and pericytes (Fig. 1A)^29,31,32^. Hb treatment significantly induced cell death in both cells in a concentration-dependent manner (Fig. 1B). To investigate whether Hb-induced cell death was related to iron and oxidative stress, the cell death assay and ROS production assay were performed with the lipid-soluble Fe^2+^ chelator, BP (Fig. 1C). Hb induced cell death and ROS over-production, and which was significantly suppressed by co-treatment with BP (Fig. 1D,E). Furthermore, a heme metabolizing enzyme, HO-1, was significantly increased after treatment with Hb in both cells (Fig. 1F). HO-1 catalyzes the conversion from heme to iron. These results suggest that the mechanism of Hb-induced ROS over-production and cell damage may be related to Fe^2+^, which is generated from Hb by HO-1.

### Fe^2+^ regent induced intracellular Fe^2+^ accumulation and cell death in both endothelial cells and pericytes

**Figure 2:**
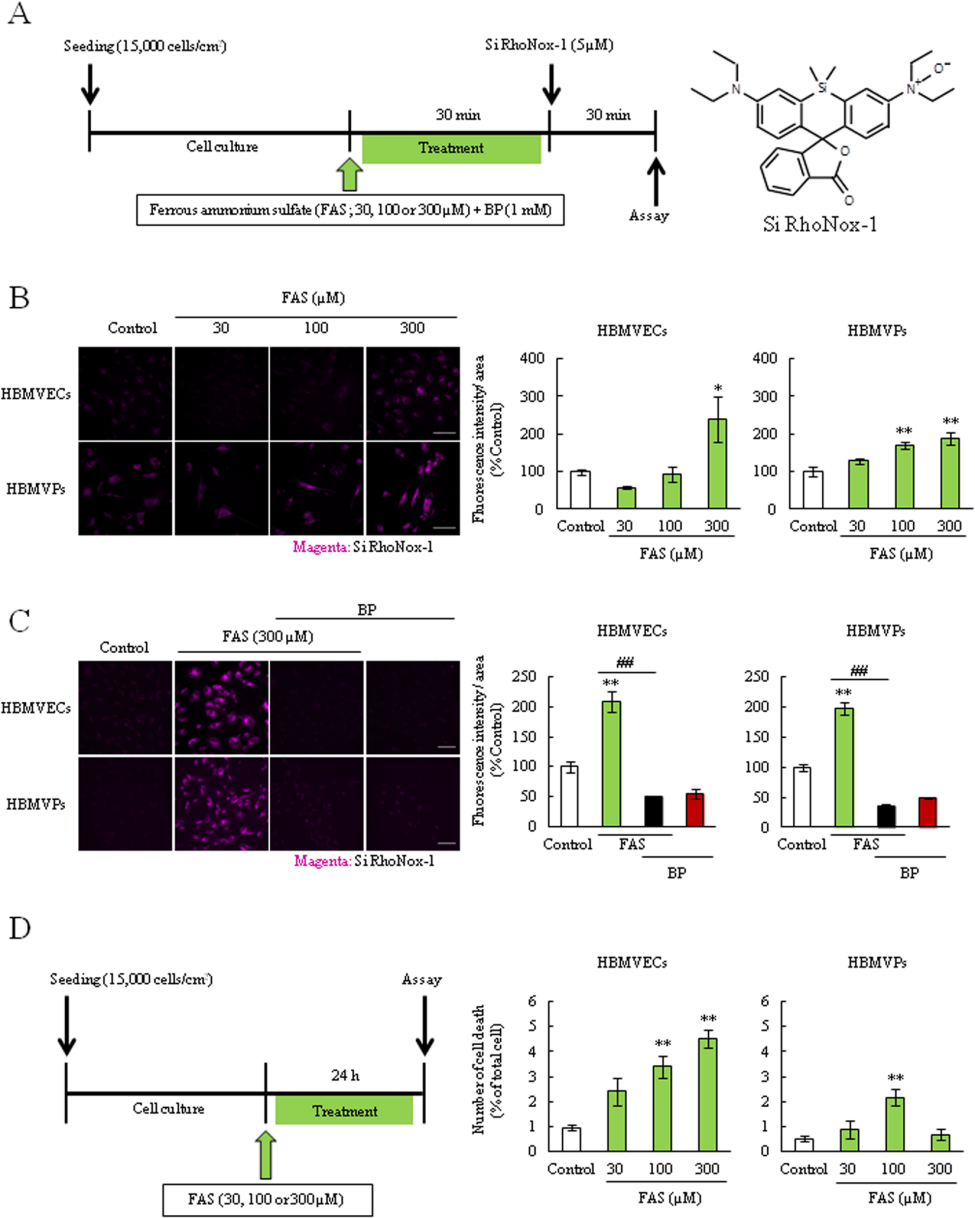
Fe^2+^ was accumulated in both endothelial cells and pericytes. (**A**) Experimental protocol of the Fe^2+^ accumulation assay and the structural formula of Si-RhoNox-1. Si-RhoNox-1 is a specific Fe^2+^-detectable fluorescent probe. Cells were incubated with ferrous ammonium sulfate hexahydrate (FAS; 30, 100 or 300 µM) for 30 min and Fe^2+^ accumulation rate was analyzed. (**B**) Intracellular Fe^2+^ accumulation assay (n = 4). (**C**) Intracellular Fe^2+^ accumulation assay upon treatment with BP (n = 4). The left images show Si-RhoNox-1 staining (5 µM; magenta). The right graphs comprise the quantitative data. (**D**) Experimental protocol of the cell death assay after FAS treatment (30, 100 or 300 µM). (**E**) The quantitative data of cell death rate (n = 6). **p < 0.01, *p < 0.05 vs. Control; ^##^p < 0.01 vs. FAS. The data was analyzed with the Dunnett’s test (**B**,**E**) or the Tukey’s test (**C**). The data are expressed as the mean ± SE. Scale bar = 100 µm.

Given the protective effect of BP in cell death and ROS production (Fig. 1), we hypothesized that Hb-induced cellular-toxicity may depend on Fe^2+^ overloading, which is a highly reactive and harmful substance. Therefore, we investigated Fe^2+^ accumulation in both cells. Firstly, we used a Fe^2+^ regent, ferrous ammonium sulfate (FAS). Cells were exposed to FAS for 30 min at a concentration of 30, 100 or 300 µM, and after treatment we monitored Fe^2+^ accumulation using the Fe^2+^ selective fluorescent probe, Si-RhoNox-1 (Fig. 2A). After incubation with FAS (300 µM), the intracellular Fe^2+^ accumulation (Fe^2+^_intra_) levels were increased and that was suppressed by BP co-treatment in both cells (Fig. 2B,C). Moreover, treatment with FAS for 24 h also significantly induced cell death (Fig. 2D). These results indicated that Fe^2+^ immediately accumulated in BBB composed cells and induced cellular toxicity.

### Intracellular Fe^2+^ accumulation was correlated to cell death in both endothelial cells and pericytes

**Figure 3:**
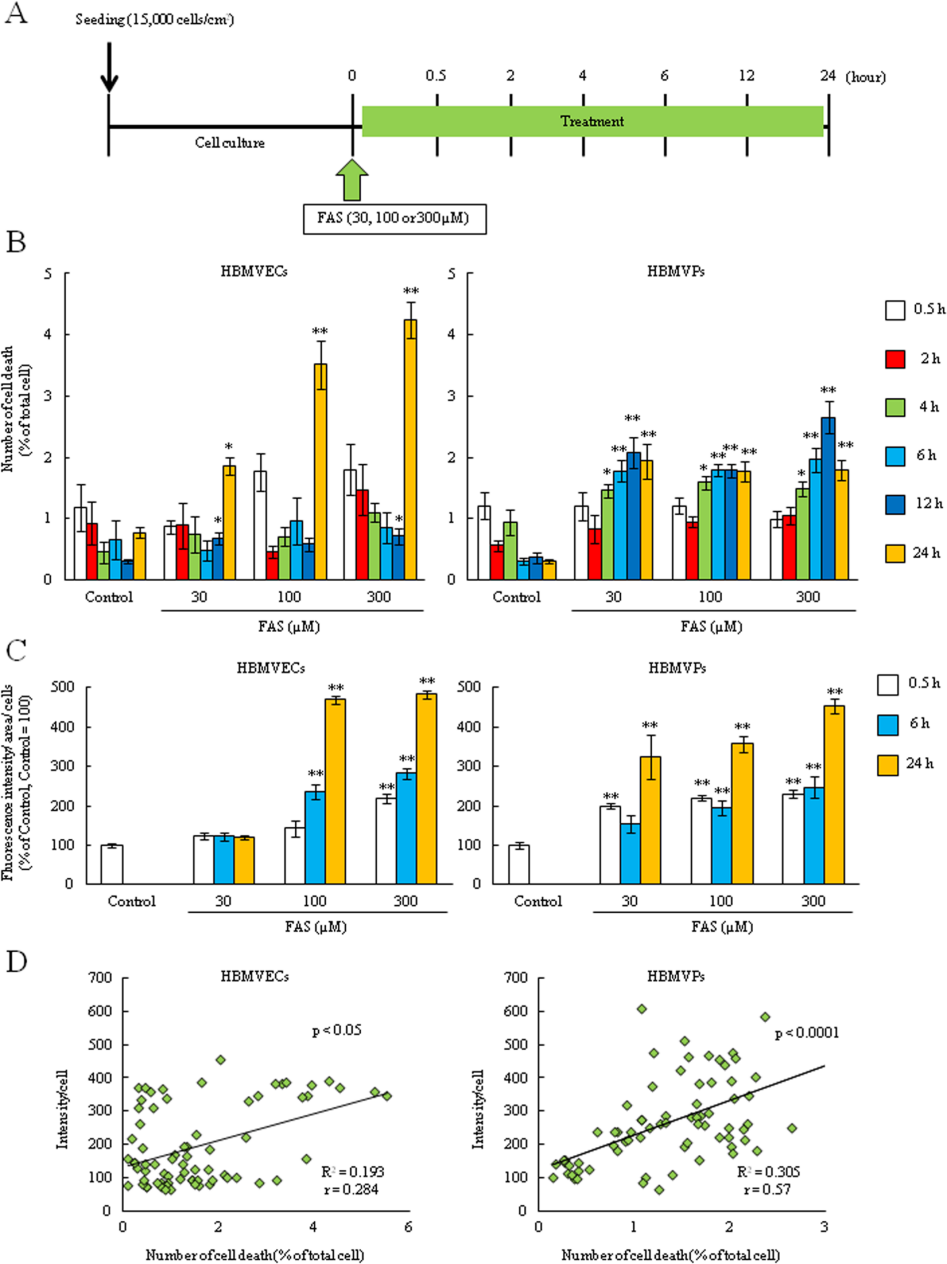
Intracellular Fe^2+^ accumulation correlated to cell death both endothelial cells and pericytes. (**A**) Experimental protocol of the time-dependently cell death and intracellular Fe^2+^ accumulation assay after FAS treatment (30, 100 or 300 µM). (**B**) Cell death rate was evaluated at 0.5, 2, 4, 6, 12 and 24 h after FAS treatment (n = 6). (**C**) Intracellular Fe^2+^ accumulation assay was performed at 0.5, 6 and 24 h after FAS treatment (n = 6). (**D**) The correlation assessment of intracellular Fe^2+^ accumulation and cell death rate (n = 72). **p < 0.01, *p < 0.05 vs. Control. The data was analyzed with the Dunnett’s test (**B**,**C**) and Spearman’s rank correlation coefficient. Scale bar = 100 µm. The data are expressed as the mean ± SE.

FAS treatment for short time induced Fe^2+^_intra_ (Fig. 2). However, the relationship between Fe^2+^_intra_ levels and cell death has been unclear. Therefore, we did the time-dependent evaluation (Fig. 3A). Cell death rate was significantly induced time-dependent manner in both cells (Fig. 3B). Although cell death rate of pericytes (0.5–2.5%) was lower than that of endothelial cells (0.5–5%), the beginning of cell death in pericytes was earlier (at 4 h). Moreover, Fe^2+^_intra_ levels were also increased time- and dose-dependent manner in both cells (Fig. 3C). As we expected, there was significantly correlation between Fe^2+^_intra_ levels and cell death (Fig. 3D). We also assessed the correlation in each time point (0.5, 6, 24 h). As results, there was significant correlation at 24 h after FAS treatment in both cells, and this correlation was observed s earlier in pericytes (6 h) (Supplemental Fig. 1A–C). These results suggested that BBB composed cells such as endothelial cells and pericytes gradually uptake the Fe^2+^ and Fe^2+^ accumulation plays a key role in cellular damage.

### Hb or hemin induced Fe^2+^ accumulation in both endothelial cells and pericytes

**Figure 4:**
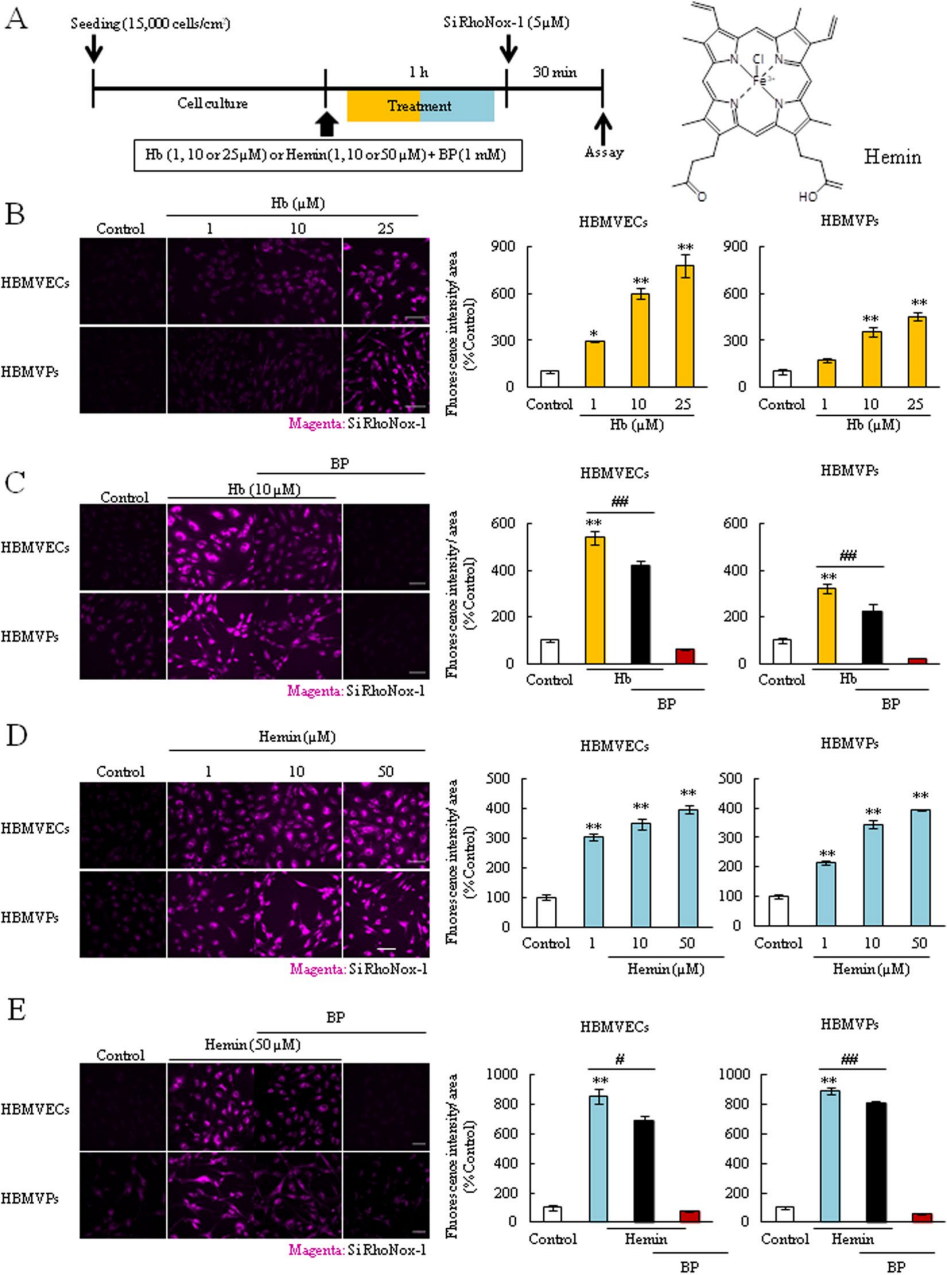
Hb or hemin treatment induced intracellular Fe^2+^ accumulation in both endothelial cells and pericytes. (**A**) Experimental protocol of the intracellular Fe^2+^ accumulation assay after Hb (1, 10 or 25 µM) or hemin (1, 10 or 50 µM) treatment, and the structural formula of hemin. (**B**,**D**) Intracellular Fe^2+^ accumulation assay (n = 3 or 4). (**C**,**E**) Intracellular Fe^2+^ accumulation assay upon treatment with BP (n = 4–6). The left images show Si-RhoNox-1 staining (5 µM; magenta). The right graphs comprise the quantitative data. **p < 0.01, *p < 0.05 vs. Control; ^##^p < 0.01, ^#^p < 0.05 vs. Hb or Hemin. The data was analyzed with the Dunnett’s test (**B**,**D**) or the Tukey’s test (C, E). Scale bar = 100 µm. The data are expressed as the mean ± SE.

Next, we performed a similar experiment by subjecting cells to Hb or hemin treatment followed by the analysis of Fe^2+^_intra_ level. Cells were exposed to Hb or hemin for 1 h (Fig. 4A). In the hemorrhagic condition, Hb emits the heme (or hemin), and heme (or hemin) is a highly reactive molecule and that generates oxidative stress-induced neuronal damage^1,13,18,33,34^. Hemin is a structurally similar to heme, and the difference between them is their valency (heme: bivalent, Fe^2+^, hemin: trivalent, Fe^3+^, Fig. 4A). At this early time point, Hb and hemin did not induce any cellular damage (data not shown). However, these treatments significantly increased the Fe^2+^_intra_ levels in a concentration-dependent manner and that were also suppressed by BP co-treatment in both cells (Fig. 4B–E). These results demonstrated that Hb or hemin were immediately converted (or released) to the Fe^2+^ form and it accumulated in BBB composed cells, which may ultimately lead to cellular-toxicity.

### Hemin induced the harmful effects on BBB composed cells and induced barrier dysfunction via iron-mediated apoptosis

**Figure 5:**
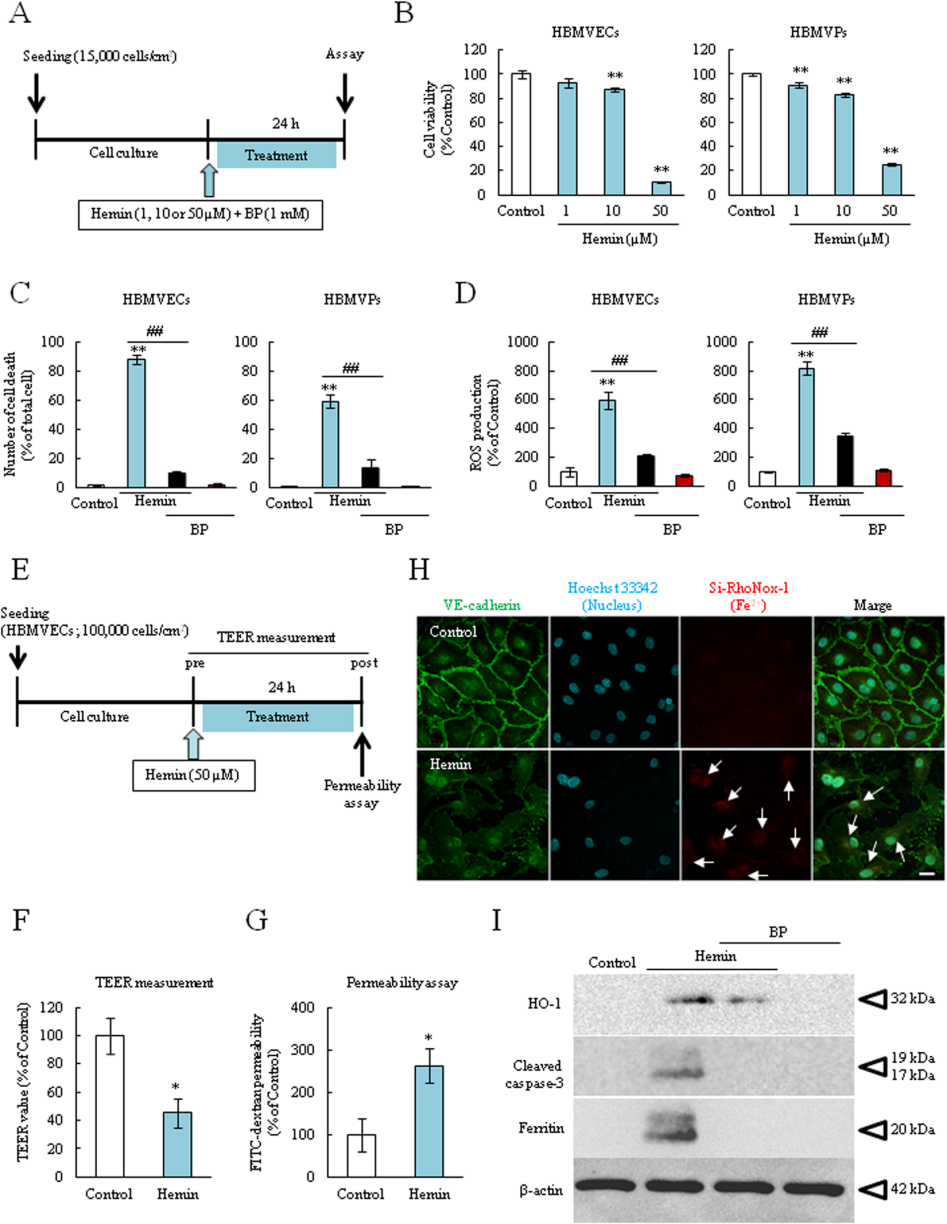
Hemin treatment induced harmful effects on both endothelial cells and pericytes, and induced endothelial barrier dysfunction. (**A**) Experimental protocol of cell viability and cell death assays. (**B**) The cell viability rate after hemin treatment for 24 hours (n = 6). (**C**) The cell death rate after co-treatment with hemin (50 µM) and BP (1 mM) for 24 hours (n = 3). (**D**) The quantitative analysis of ROS production after hemin with BP co-treatment (n = 5–6). The ROS production rate was corrected by the number of living cells. (**E**) Experimental protocol of TEER value measurement and FITC-dextran permeability assay after hemin treatment (50 µM). (**F**) The obtained TEER values at 24 h after hemin treatment (n = 4). (**G**) Permeability rate (n = 4). (**H**) The representative fluorescent images of endothelial junction after performing TEER and FITC-dextran assay. The following fluorescent probes were utilized to detect different cellular components: VE-cadherin (anti-VE-cadherin antibody, green), nucleus (Hoechst 33342, blue), and Fe^2+^ (Si-RhoNox-1, red). (**I**) The each representative band images by western blotting analysis. HO-1, the heme metabolizing enzyme, cleaved caspase-3, an apoptosis marker, ferritin, an iron storage protein, β-actin, the loading control. **p < 0.01, *p < 0.05 vs. Control; ^##^p < 0.01, ^#^p < 0.05 vs. Hemin. The data was analyzed with the Dunnett’s test (**B**), the Tukey’s test (**C**,**D**) or the Student’s *t* test (**F**,**G**). Scale bar = 20 µm. The data are expressed as the mean ± SE.

In order to elucidate the mechanism of Hb-mediated cellular toxicity, we evaluated the effects of hemin on both cells. Firstly, we investigated the harmful effects of hemin in both cells (Fig. 5A). Treatment with 10 or 50 µM hemin for 24 h significantly reduced cell viability in both cells, and 1 µM hemin also reduced cell viability in pericytes (Fig. 5B). Furthermore, high concentration of hemin (50 µM) induced severe cell death and ROS over-production, and which were suppressed by BP co-treatment (Fig. 5C,D). These results suggested that the main factor contributing Hb-induced cellular toxicity may be hemin, and the bound iron may be involved in the cell damage mechanism. Next, we evaluated the endothelial barrier function by assessing the trans-endothelial electrical resistance (TEER) value, fluorescein isothiocyanate (FITC)-dextran permeability rate and immunostaining of vascular endothelial-cadherin (VE-cadherin) (Fig. 5E). Normal cell-cell junctions limit ion and large molecular permeability, and the TEER value and permeability rate are often altered by pathological conditions. After hemin (50 µM) treatment for 24 h, the TEER value was significantly decreased and FITC-dextran permeability rate was significantly increased compared to normal condition (Fig. 5F,G). Especially, hemin treatment disrupted the VE-cadherin integrity and increased the Fe^2+^_intra_ level (Fig. 5H). Furthermore, the expression of several proteins including HO-1 (heme metabolize enzyme), cleaved caspase-3 (an apoptosis marker), and ferritin (an iron storage protein marker) were altered after hemin treatment for 24 h. Importantly, BP co-treatment suppressed the expression of proteins up-regulation (Fig. 5I). As with Hemin treatment, Hb also induced endothelial barrier dysfunction and protein alteration (Supplemental Figs 2, 3). These results suggested that Hb or hemin may be an important factor in endothelial barrier dysfunction in secondary brain injury after ICH, and Fe^2+^ accumulation may be strongly related to this phenomenon.

### Hemin injection induced brain damage in an *in vivo* mice model

**Figure 6:**
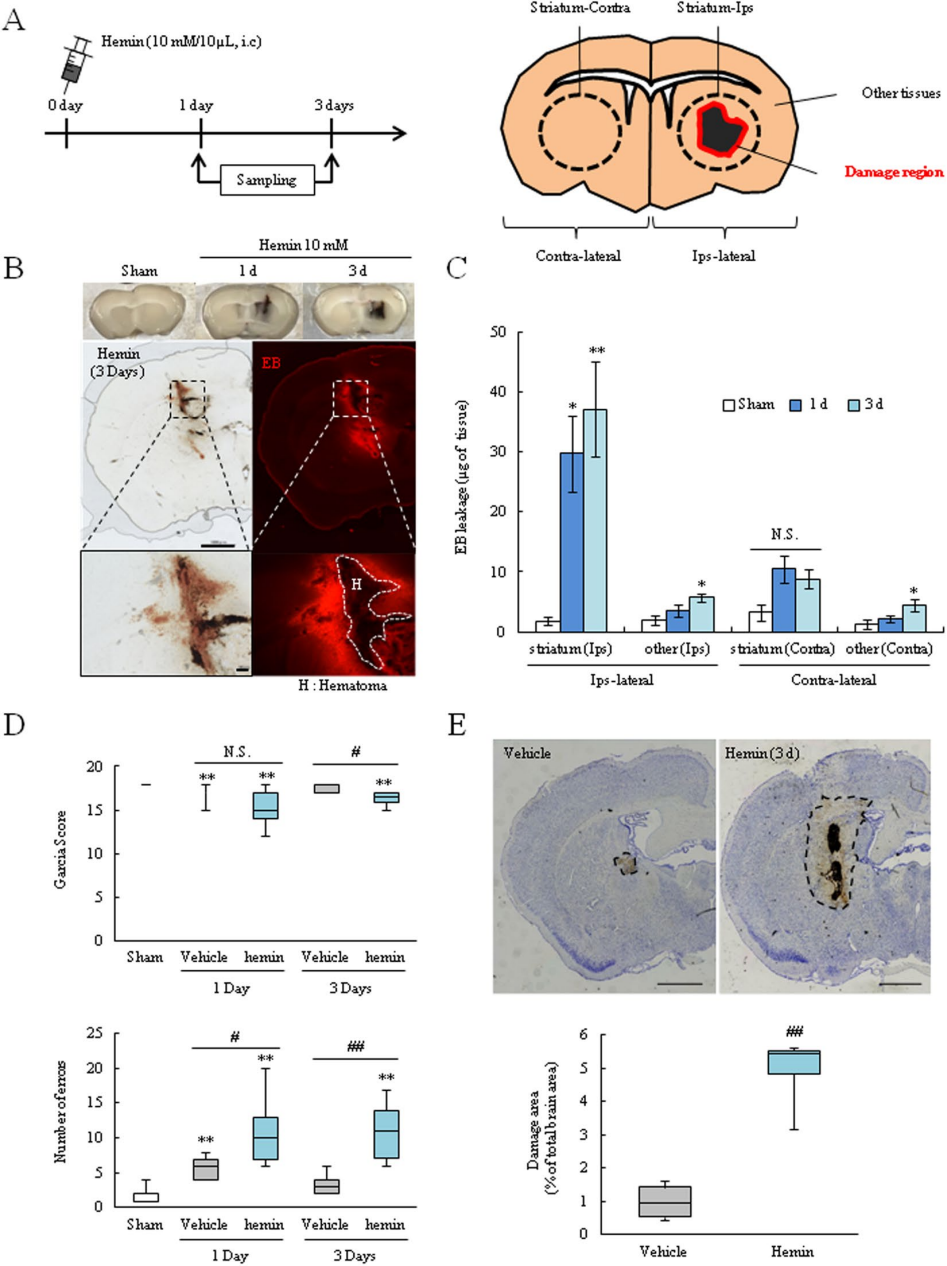
Intracerebral injection of hemin induced BBB damage *in vivo*. (**A**) Experimental protocol of the hemin injection model. The schema of the mouse brain after hemin injection. Hemin was administered into the striatum region. The injection site (striatum) and other tissue in both the ipsilateral and contralateral regions were harvested. (**B**) The upper images show the representative brain slices after hemin injection. The lower images depict the Evans blue leakage in brain sections and the hematoma region (**H**) is shown. (**C**) The graph represents the quantitation of Evans blue leakage (µg of brain tissue), which indicates BBB permeability (Sham, n = 5; Hemin 1 day, n = 9; Hemin 3 day, n = 9). (**D**) Behavior test data. The upper panel shows the Garcia test score and the lower panel shows the number of errors in a grid-walking test (Sham, n = 8; Vehicle 1 day, n = 5; Vehicle 3 day, n = 5; Hemin 1 day, n = 9; Hemin 3 day, n = 7). (**E**) Neuronal damage assay. The upper panel shows the representative brain images which were stained by cresyl violet and the lower graph represents the quantitation of the damage area/total brain area (Vehicle, n = 4; Hemin, n = 4). Scale bar = 1 mm. **p < 0.01, *p < 0.05 vs. Sham; ^##^p < 0.01, ^#^p < 0.05 vs. Vehicle; ^$^p < 0.05 vs. Hemin. The data was analyzed with the Tukey’s test (**C**), the Mann-Whitney *U*-test (**D**), or the Student’s *t* test (**E**). The data are expressed as the mean ± SE (**C**) or SD (**D**, **E**).

According to our *in vitro* experiments, exposure to FAS, Hb or hemin damaged BBB composed cells. Of the three toxic agents, hemin exposure results in more severe injury. Hence, we performed *in vivo* experiments to investigate whether hemin induces BBB damage (Fig. 6A). The leakage of evans blue (EB), which indicates BBB hyper-permeability, occurred in the perihematoma region (Fig. 6B). This leakage was significantly increased at 1 and 3 days after surgery. The striatum region (peri-injection area) had significantly more EB dye compared to other tissues (Fig. 6C). The neurological function analysis demonstrated that the hemin injection group significantly aggravated neuronal deficit symptoms compared with the other group at 3 days after surgery (Fig. 6D). The neuronal damage area was also significantly increased in the hemin injection group compared to the vehicle group as assessed by cresyl violet staining (Fig. 6E). These results suggested that hemin induced BBB damage and leakage blood components which may lead to neurological symptom.

### Hemin injection altered BBB integrity and accumulated iron in both endothelial cells and pericytes

**Figure 7:**
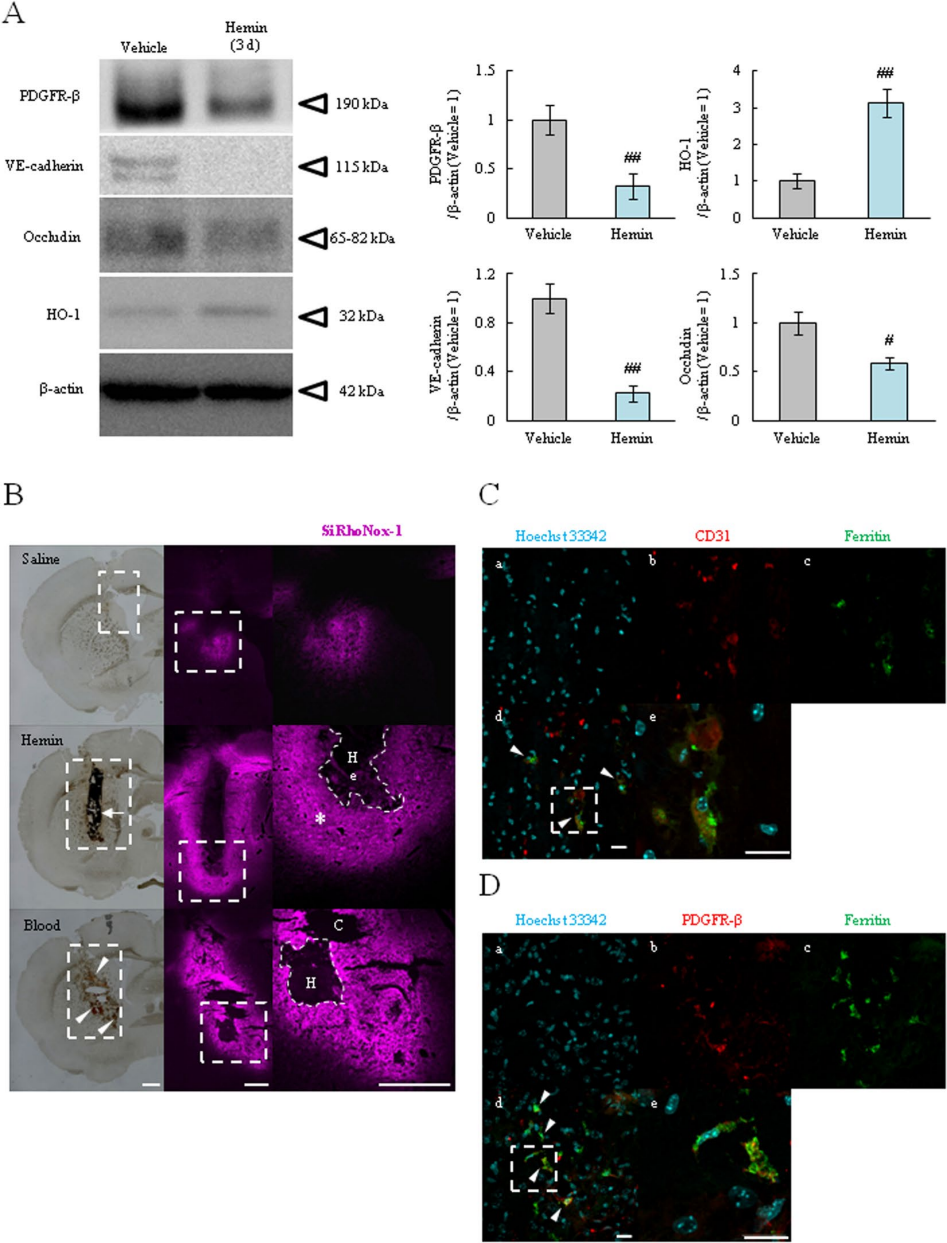
Intracerebral injection of hemin induced Fe^2+^ accumulation in both endothelial cell and pericyte at peri-hematoma region. (**A**) Western blot analysis of HO-1, VE-cadherin, PDGFR-β and occludin in the damaged region 3 days after hemin injection (Vehicle, n = 7; Hemin, n = 7). (**B**) The detection of Fe^2+^ accumulation at 3 days after hemin injection using Si-RhoNox-1. The groups comprise the following: upper is saline injection (Vehicle), middle is hemin injection (Hemin) and lower is autologous blood injection (Blood). In the brightfeild images, the arrow (black area) shows remnant hemin and the arrow heads (red area) delinate the hemorrhagic area. In the fluorescent images, the dod line square indicates the enlarged area. The Si-RhoNox-1 staining (magenta) is shown. [He] Remnant hemin, [H] the hematoma, and [**C**] the cavity portion of the brain are shown (Vehicle, n = 4; Hemin, n = 4; Blood, n = 3). Scale bar = 500 µm. (**C**) Representative immunofluoroscense staining at peri-hematoma region (* mark in B). [a] Hoechist 33342 (blue; nucleus), [b] CD31 (red; the marker of endothelial cell), [c] Ferritin (green; the marker of iron), [d] the maged image and [e] enlarged image of dod line square in the marged image (n = 3). (**D**) Representative immunofluoroscense staining. [b] PDGFR-β (red; the marker of pericyte) (n = 3). Scale bar = 20 µm. ^##^p < 0.01, ^#^p < 0.05 vs. Vehicle. Arrowheads indicate merged cells. The data was analyzed with the the Student’s *t* test (**A**) and expressed as the mean ± SE.

We found that hemin injection induced BBB injury and hyper-permeability in mouse model (Fig. 6). Based on these results, we investigated the hemin effects on BBB composed cells by western blotting and immunohistological assessment at 3 days after surgery. As results, VE-cadherin and occludin were decreased while the expression of HO-1 was increased in the hemin injection group compared the vehicle group. In addition, the pericyte marker, platelet-derived growth factor receptor-β (PDGFR-β), was also decreased (Fig. 7A).

Next, to investigate Fe^2+^ accumulation in the *in vivo* mice model, we performed experiments similar to *in vitro* studies utilizing the specific probe, Si-RhoNox-1. Figure 7B shows that the brains in saline injection group (Vehicle) exhibited negligible fluorescence in a small area; this is thought to be derived from needle damage. On the other hands, the hemin injection group (Hemin) exhibited robust fluorescence in an area was larger than the saline group, and fluorescence was detected in the peri-injection area (arrow). We also evaluated in an autologous blood injection model. In this model, the fluorescence pattern was similar to that obtained with hemin injection, and fluorescence was also detected in the perihematoma region (arrowhead). Moreover, to identify the iron accumulated cells in peri-hematoma region (* in Fig. 7B), we used CD31 (an endothelial cell marker), PDGFR-β and Ferritin antibodies. We detected the merged cells (Ferritin^+^-CD31^+^ or PDGFR-β^+^ cells) in peri-hematoma region. These results indicated that hemin releases Fe^2+^ from the hematoma to surrounding tissue and its accumulated in BBB composed cells, which ultimately induced BBB disruption via disassembling tight junction proteins at 3 days after ICH. Therefore, this iron release may be crucial for the induction of secondary brain injury.

### An iron chelator ameliorates neurological dysfunction in the hemin injection ICH mice model

**Figure 8:**
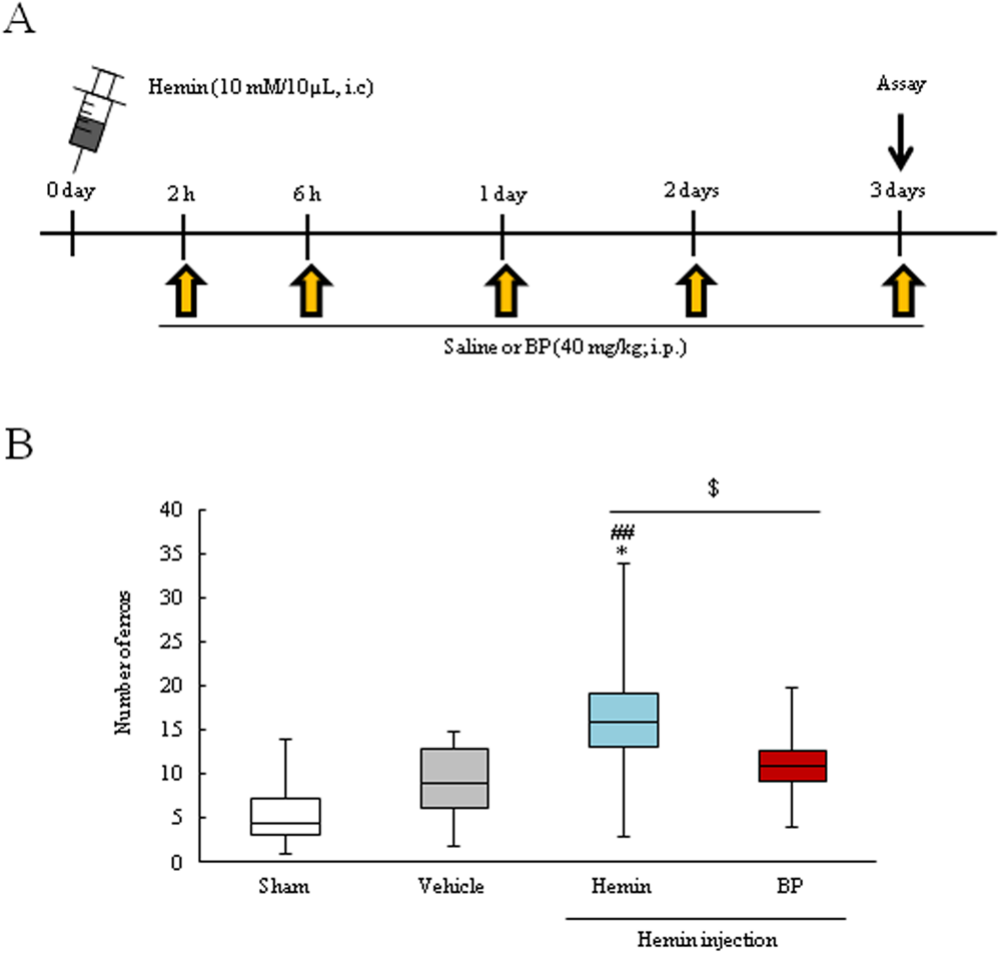
The protective effect of an iron chelator in *in vivo* hemin model. (**A**) Experimental protocol of BP treatment in the hemin injection model (Sham, n = 3; Vehicle, n = 9; Hemin, n = 9; BP, n = 9). BP or saline was administered 5 times after hemin injection (2 h, 6 h, 1 day, 2 days and 3 days). (I) Behavior test data. The graph shows the number of errors in grid-walking test. *p < 0.05 vs. Sham; ^##^p < 0.01 vs. Vehicle; ^$^p < 0.05 vs. Hemin. The data was analyzed with the the Mann-Whitney *U*-test (**B**) and expressed as the mean ± SD.

The *in vitro* results showed that BP, an iron chelator, may have protective effects against Hb or hemin-induced cellular damage. In addition, Fe^2+^ accumulation was detected in the perihematoma region in the hemin injection ICH mice model. Therefore, we investigated the effects of BP administration in this model (Fig. 8A). 5 times administration of BP significantly attenuated neurological deficit (Fig. 8B). This result suggested that Fe^2+^accumulation may be strongly related to hemin-induced neuronal damage, and treatment with an iron chelator may be effective for ICH patients.

## Discussion

In the present study, we demonstrated the following: (1) Hb or hemin each independently induced cell death and accelerated ROS production in BBB formation cells (i.e. endothelial cells and pericytes); (2) the Fe^2+^_intra_ accumulation levels were immediately increased in both cells after treatment with FAS, Hb or hemin; (3) there was correlation between Fe^2+^_intra_ accumulation levels and cell death; (4) Hemin or Hb each independently induced endothelial barrier dysfunction and hemin disrupted the VE-cadherin integrity; (5) after hemin injection into brain parenchyma, BBB permeability was aggravated via increasing of HO-1 and degradation of cell-to-cell junction proteins and pericytes loss; (6) Fe^2+^ accumulated in both endothelial cells and pericytes at the peri-injection region 3 days after hemin injection and (7) the iron chelator significantly improved neurological deficit induced by hemin injection in an ICH model. In summary, these results indicated that the chelation of intracellular Fe^2+^ in BBB composed cells may be a useful treatment strategy for the prevention of secondary brain injury following ICH.

In the ICH condition, blood components induce brain tissue damage via oxidative stress^1,10^. In the present study, we focused on BBB composed cells and showed that human Hb treatment significantly induced cell death and ROS over-production in human brain endothelial cells and pericytes and which were suppressed by treatment with t a lipid-soluble iron chelator, BP (Fig. 1B,D,E). Considering to the effects of BP, the mechanism of Hb-induced cell death may be related to iron and ROS over-production. Previous reports have shown that after Hb is dissolved in water, it is immediately converted to heme (or hemin), and subsequently decomposed to Fe^2+^ or Fe^3+^ by HO-1/2^13,33,35^. These irons catalyze ROS production via Fenton reaction, and induce oxidative stress damage^10,21,36^. In both endothelial cells and pericytes, the expression of HO-1, an inducible isoform, was significantly increased after Hb treatment (Fig. 1F). HO-1 is strongly linked to oxidative stress, and previous reports have demonstrated that HO-1 is a key factor in aggravating the ICH condition^37,38^.

Based on these findings and previous studies which investigated the Hb-induced damage to neuron^14,34,39^, we hypothesized that Fe^2+^ is a crucial factor in Hb-induced cellular damage. In this study, to investigate Fe^2+^_intra_, we used the novel specific probe Si-RhoNox-1 which has adequate stability and does not react with reducing substances. Therefore, this probe can exclusively detect Fe^2+^ but not Fe^3+^ ^40^. As results, the Fe^2+^_intra_ levels after treatment of iron contain substances were rapidly increased and there was the correlation between the Fe^2+^_intra_ levels and cell death, which BP inhibited this increase in both cells (Figs 2–4, Supplemental Fig. 1). Interestingly, this probe showed fluorescence after hemin treatment although hemin contains Fe^3+^ (Fig. 4D,E). Therefore, we found that the iron form in hemin is instantaneously converted from Fe^3+^ to Fe^2+^ after intracellular import. Previous reports show that Fe^3+^ normally binds to the iron transport protein transferrin before cellular uptake^21,41^. This complex is then disassembled and Fe^3+^ is reduced to Fe^2+^ via ferrireductase. After the reduction reaction, the released Fe^2+^ accelerates ROS production and induces mitochondrial dysfunction^16,36,42^. In fact, recent studies have shown that hemin exposure induces mitochondrial dysfunction, ferroptosis. Moreover, this molecule also induces the neuronal cell death via activating matrix metalloprotease-9 and toll like receptor 2^16–18,34^. In the present study, hemin treatment increased not only the Fe^2+^_intra_ levels but also dramatically induced the cell death in both endothelial cells and pericytes, particularly cell death seemed to be terrible compared to FAS or Hb treatment (Fig. 5B). We also found that treatment with Hb or hemin induced the endothelial barrier dysfunction and hemin specifically induced the disruption of VE-cadherin integrity and altered several proteins expression (Fig. 5G–I and Supplemental Figs 2 and 3). Ferritin and HO-1 are strongly linked to iron metabolism, therefore, this barrier damage may be related to Fe^2+^_intra_ accumulation and apoptosis. Collectively, these findings indicated that Fe^2+^ accumulation may strongly contribute to the mechanism of Hb or hemin-induced both cellular damage and disrupting endothelial junction integrity in ICH pathology, especially hemin may be key factor.

Our *in vivo* studies demonstrated that hemin induced BBB damage and neuropathy (Fig. 6C–E). These findings are likely to a previous report, which shows that hemin injection induced the brain damage^17^. However, the detailed effects of hemin on the BBB are still unclear. Therefore, we investigated the BBB component markers such as VE-cadherin, occludin, and PDGFR-β based on our *in vitro* results. These proteins were decreased in the perihematoma region at 3 days after surgery (Fig. 6A). Similar to *in vitro* results, vascular cells were injured, which suggested a weakening of the BBB integrity. We also found that HO-1 was increased after hemin injection (Fig. 6F). In the ICH condition, the effects of HO-1 can have favorable or unfavorable ramifications. For example, HO-1 deletion protects against iron-induced injury and HO-1 increase exacerbates brain injury after ICH^37,38^. On the other hand, the NF-E2-related factor 2/HO-1 pathway activators such as nicotinamide and (−)-epicatechin attenuated brain injury after ICH^11,43,44^. Interestingly, a recent study showed that the effects of HO-1 varies with time in the ICH model; HO-1 plays a harmful role in the acute phase (1–3 days), but changes to a neuroprotective effect in the chronic phase (28 days)^45^. Since our experiments were conducted in the acute phase, HO-1 might accelerate brain damage via converting heme to iron. However, this hypothesis limits within expectation and the detrimental effect of HO-1 has been still controversial^46^. We confirmed that hemin injection or autologous blood injection induced Fe^2+^ accumulation in the peri-injection region (Fig. 7B). This is the first report documenting the Fe^2+^ accumulation around a hematoma in ICH models such as the hemin injection model and the autologous injection model. Moreover, iron accumulation was localized in endothelial cells and pericytes(Fig. 7C,D), which similar to *in vitro* experiments (Figs 2, 4). These *in vivo* results indicated that hemin has harmful effects which induced intracellular iron accumulation and BBB damage via decreasing the junction proteins and pericytes.

Iron is an extremely important factor for maintaining homeostasis, but our study suggests that it occasionally generates harmful effects, especially in stroke cases^20^. Recently, ferroptosis, (a novel type of programmed cell death) has been linked to stroke damage^9,18^. This cell death pathway is dependent on iron. Thus, iron chelators (deferoxamine) and hemoglobin-related scavenger proteins such as hemopexin and haptoglobin are expected to be useful for the prevention of secondary brain injury after ICH in clinical state^24–,26,47,48^. Based on *in vitro* results, we investigated the effect of BP administration on the hemin injection ICH model. The protective effect of BP against ICH has been controversial yet; Whereas Wu H *et al*. indicated that BP attenuated the brain damage and improved the functional outcome in an experimental ICH model^49^. on the other hands, Caliaperumal J *et al*. reported contradict results that BP has no effect on ICH condition^50^. In present study, 5 times BP administration improved neuronal deficits at 3 days after hemin injection (Fig. 8B). Thus, these results support our hypothesis that the iron molecule may contribute to secondary brain injury.

In conclusion, our findings indicated that Hb or hemin each independently induced damage to both endothelial cells and pericytes via intracellular Fe^2+^ accumulation. *In vitro* and *in vivo* studies revealed that an iron chelator protected against the cell damage. Hemin induced endothelial barrier dysfunction and disrupted junction integrity *in vitro*, and induced BBB hyper-permeability in an *in vivo* ICH mouse model. Taken together, these results suggested that modulating or chelating intracellular Fe^2+^ in BBB component cells may be useful for the prevention of secondary brain damage after ICH.

## Materials and Methods

### Cell culture

Human brain microvascluar endothelial cells (HBMVECs) (DS Pharma Biomedical, Osaka, Japan) and pericytes (HBMVPs) (Primary Cell, Kyoto, Japan) were cultured dishes in Endothelial cells Basal Medium (EBM^TM^−2 Bullete kit^TM^; Lonza, Basle, Switzerland) or Pericyte medium (ScienCell, Corte Carlsbad, CA, USA), each medium contains 10% fetal bovine serum (FBS; Thermo Scientific, Waltham, MA, USA) and several supplements. Both cells were respectively incubated at 37 °C under a humidified 5% CO_2_ atmosphere until they reached confluence. Cells were seeded on the each plate, which was coated by collagen (Cellmatrix^®^; Nitta Zelatin Inc., Osaka, Japan) or poly-L-lysine (2 µg/cm^2^; ScienCell). Medium was changed every 2 days.

### Cell death and viability assay

HBMVECs or HBMVPs were seeded into a 96-well plate (BD Biosciences, Franklin Lakes, NJ, USA) and incubated to reach confluent (seeding density; 15,000 cell/cm^2^). Human Hb (Molecular weight, 64,500; Sigma-Aldrich, St. Louis, Missouri, USA) was dissolved into sterilized water and diluted with culture medium (1, 10, 25 µM). Hemin (Molecular weight, 651.95; Tokyo Chemical Industry Co., Ltd, Tokyo, Japan) was dissolved into dimethyl sulfoxide (DMSO; Nacalai tesque, Kyoto, Japan) and diluted with culture medium (1, 10, 50 µM). A final concentration of DMSO was 0.1%, which did not damage the cells. In addition, a Fe^2+^ reagent, ferrous ammonium sulfate hexahydrate (FAS; molecular weight, 392.14; Wako Pure Chemicals, Osaka, Japan) was dissolved into sterilized water and diluted with culture medium (30, 100, 300 µM). Cells were incubated with Hb for 4 h or hemin and FAS for 24 h (Cell death was evaluated at 0.5, 2, 4, 6, 12 and 24 h in FAS treatment) (Figs 1A,C, 2D, 3A and 5A), that protocol was decided based on previous reports^12,14^. After treatment, Hoechst 33342 and propidium iodide (PI) were added to culture medium for 15 min at the final concentrations of 8.1 µM and 1.5 µM, respectively. Images were collected using an Olympus IX70 inverted epifluorescence microscope (FV10i; Olympus, Tokyo, Japan). The total number of cells was counted and the rate of PI-positive cell number was calculated in a blind manner by a single observer (T. I.).

Cell viability was measured by using Cell Counting Kit-8 (CCK-8; Dojin Kagaku, Kumamoto, Japan). Both cells were incubated with hemin (1, 10, 50 µM) for 24 h. Subsequently, CCK-8 reagent was added to each well at 10 µL and incubated for 3 h. At 0 h and 3 h, the absorbance was measured by using microplate reader at 450 nm^29,31^.

### ROS production assay

In order to evaluate intracellular ROS production after Hb or hemin treatment, we used CM-H_2_DCFDA (Thermo Fisher Scientific). Treated cells were incubated with CM-H_2_DCFDA for 30 min at 37 °C. Fluorescent signals were measured by using the Varioskan Flash 2.4 microplate reader (Thermo Fisher Scientific) at ex/em: 495/527 nm. ROS production rate was corrected by the number of live cells^31^.

### Intracellular bivalence iron accumulation assay

Fe^2+^_intra_ levels were evaluated by using Si-RhoNox-1, which we developed fluorescence probe to detect Fe^2+^ ^40^. The structural formula of this probe is described in Fig. 2A. Both cells were seeded into CELLview^TM^ glass bottom dish (4-wells, 35 × 10-mm; Greiner Bio-One Int., Kremsmünster, Austria), and incubated to reach sub-confluent (seeding density; 15,000 cell/cm^2^). The loading time of Hb or hemin was for 1 h, FAS was loaded for 0.5 h (FAS was incubated for 0.5, 6 and 24 h in time-dependently assessment). After incubation with them, culture medium was washed by phosphate buffered solution (PBS; Wako Pure Chemicals) and exchanged for Hanks’ Balanced Salt Solution (HBSS; Thermo Fisher Scientific) containing Si-RhoNox-1 (5 µM). Subsequently, medium was exchanged and then observed the fluorescence under a confocal microscope at 575 nm (excitation: ex) and 660 nm (emission: em). The protocol was described in Figs 2A, 3A, and 4A.

### Iron chelator treatment *in vitro* study

To evaluate the relation of Fe^2+^ in Hb or hemin damage, we used 2,2′-bipyridil (BP; molecular weight, 156.19) as the iron chelator, which we synthesized. The structural formula of BP was described in Fig. 1C. Firstly, BP was dissolved in DMSO (1 M) as stock solution, and then diluted with PBS. BP was added to culture medium with Hb, hemin, or FAS. (The final concentration of BP was 1 mM, and the final concentration of DMSO was 0.1%). The effects of co-treatment with BP were evaluated by cell death assay, ROS production assay, and Fe^2+^ accumulation assay. In all experiments, there was no cellular damage in BP only treatment group.

### Evaluation of endothelial barrier function

Endothelial barrier function was evaluated by trans-endothelial electrical resistance (TEER) and FITC-dextran permeability rate as described previously^29^. HBMVECs were seeded into Transwell^®^ inserts (upper wells), and these inserts were put in 24-well plate (Corning, Inc., Canton, NY, USA), and then incubated to reach confluent to form sufficient cell-to-cell junctions (seeding density; 100,000 cell/cm^2^). TEER value was measured at pre- and post-treatment of Hb (10 µM) or hemin (50 µM) by using an Epithelial Volt-Ohm Meter (Millicell, ESR-2, Millipore, Merck Millipore Co., Darmstadt, Germany) and a cup-shaped electrode (Endohm-6, World Precision Instruments, Sarasota, FL, USA) (Fig. 5A and Supplemental Fig. 2A).

After measuring TEER value, the medium in upper wells was exchanged medium containing FITC-dextran (1 mg/kg, Sigma-Aldrich) and incubated for 1 h. After that, the medium diluted by PBS and diluted medium was placed in 96-well plate. Then, absorbance was measured by using microplate reader at 485/530 nm.

After FITC-dextran assay, the medium in upper wells was exchanged medium containing Si-RhoNox-1 (5 µM) and incubated for 1 h. Thereafter, we performed the immunostaining analysis. Cells were fixed in 4% paraformaldehyde (PFA) in 0.1 M phosphate buffer (PB; pH 7.4) for 15 min at room temperature, and incubated with 0.3% Triton-X-10/PBS to increase permeability for 10 min at room temperature. After blocking with 5% goat serum/PBS for 30 min, and incubated overnight at 4 °C with anti-vascular endothelial-cadherin (VE-cadherin) rabbit polyclonal antibodies (1:400, Abcam, Cambridge, UK). After washes, cells were incubated for 1 h with secondary goat anti-rabbit AlexaFluor^®^ 488 (Thermo Fisher Scientific). After washes, cells were counterstained with Hoechst 33342. Images were obtained by confocal microscope. The wavelength of VE-cadherin was at ex/em: 485/530 nm^29,31,32^.

### *In vitro* Western blotting analysis

Western blotting method has been reported previously^29^. Both cells were seeding on 12-well plate (seeding density; 15,000 cells/cm^2^). After having reached confluent, Hb or hemin was added into culture medium and incubated for 4 h or 24 h. Subsequently, the cells were washed with PBS, and then lysed in buffer (50 mM Tris–hydrogen chloride (HCl), pH 8.0, containing 150 mM sodium chloride (NaCl), 50 mM ethylene diamine tetraacetic acid (EDTA), 1% Triton X-100, and a protease/phosphatase inhibitor mixture) containing a protease inhibitor and phosphatase inhibitor cocktails (Sigma-Aldrich, St. Louis, Missouri, USA). The lysate was centrifuged and the supernatant was harvested. Using protein assay kit (Thermo Scientific), protein concentration was determined. A 2-µg protein sample was subjected to electrophoresis on a 5% to 20% gradient sodium dodecyl sulfate (SDS)-polyacrylamide gel (SuperSep Ace; Wako Pure Chemicals), and the separated proteins were subsequently transferred to a polyvinylidene difluoride membrane (Immobilon-P; Merck Millipore Corp, Billerica, MA, USA). For western blotting, the following primary antibodies were used: cleaved caspase-3 (1:1000, Cell Signaling Technology, Beverly, MA, USA), HO-1 (1:200; Santa Cruz, Biotechnology, CA, USA), Ferritin (1:1000, Abcam, Cambridge, UK), β-actin (1:2000; Sigma-Aldrich). Used antibodies were summarized in Table 1.

**Table 1:** The used antibodies in present study.

Target	Manufacturer	Code	Dilution	Clonallty	Reactivity
HO-1	Santa Cruz	sc-01789	1/200	Polyclonal	Rabbit
Cleaved caspase-3	Cell signaling Technology	9664S	1/1000	Polyclonal	Rabit
Ferritin	Abcam	ab75973	WB:1/1000 IHC: 1/100	Monoclonal	Rabbit
VE-cadherin	Abcam	ab33168	WB:1/1000 IHC: 1/400	Polyclonal	Rabit
Occludin	Abcam	ab64482	1/2000	Polyclonal	Rabit
PDGFR-β	Santa Cruz	WB: sc-432 IHC: sc-19995	WB:1/200 IHC: 1/100	WB: Polyclonal IHC: Monoclonal	WB: Rabbit IHC: Mouse
β-actin	Sigma-Aldrich	A2228	1/2000	Monoclonal	Mouse
CD31	BD Biosciences	550274	1/100	Monoclonal	Mouse

Immunoreactive bands were visualized using ImmunoStar LD (Wako Pure Chemicals). Band intensities were measured using a Lumino imaging analyzer (LAS-4000; Fujifilm, Tokyo, Japan). Expression level differences were analyzed with Image-J software version 1.43 h (NIH, Bethesda, MD, USA) by measuring band intensities.

### Animals

Male ddY mice (8 weeks old; body weight, 35–40 g; Japan SLC Ltd., Hamamatsu, Japan) were used in all *in vivo* experiments. All animal protocols were approved, and were conducted in accordance with the Animal Research: Reporting *in Vivo* Experiments guidelines and the Institutional Animal Care and Use guidelines of the Experimental Committee of Gifu Pharmaceutical University, Japan (Ethic notes. 2017-001, 134, 135, 223, 2018-008, 009). Animals were housed at 24 °C ± 2 °C under a 12 h light-dark cycle. Food and water were freely available to all animals. Operator (T. I.) and observer (S. I.) were blinded to the treatment status of the animals in each experiment.

### *In vivo* hemin injection model

Mice were anesthetized with 2.5% to 3.0% isoflutane (Mylan Inc., Canonsburg, PA, USA) in 70% N_2_O/30% O_2_, which was delivered using a facemask with an animal general anesthetized machine (Soft Lander, Sin-ei Industry Co., Ltd., Saitama, Japan). After scalp incision and a small burr hole was drilled, mice were gently injected with saline or hemin (10 mM in PBS containing 1% DMSO) into striatum (2-mm lateral to the bregma and a depth of 3.5-mm) at a rate of 1 µL/min by using Hamilton syringe (Hamilton, 7000 series syringes, Hamilton Company, Inc., Reno, NY, USA), the injection volume was 10 µL. The needle was left for 5 min, and removed over 3 min to minimize backflow. Sham-operated mice underwent the incision. After the surgery, mice were housed under the preoperative conditions until further experiments were performed (Fig. 6A)^17,27,29^.

In this study, we used 114 mice and excluded 5 mice from samples according to following exclusion criteria; injected materials such as hemin and autologous blood were leaked to brain surface. No mice were died in this study.

### BBB permeability assay

BBB permeability was evaluated by the extravasation of Evans blue (EB, Wako Pure Chemicals), a marker of albumin penetration as described previously^30^. EB dye (2% in PBS, 4 ml/kg) was administered though the tail vein at 1 or 3 days after surgery. Mice were euthanized 1 h after the EB dye injection and transcardially perfused with cold saline for 2 min at room temperature. Removed brains were divided into hemisphere, these were separated to striatum and other region and measured tissue weight respectively. The samples were rinsed in PBS (250 µL) and homogenized, thereafter, 50% trichloroacetic acid (250 µL; Wako Pure Chemicals) was added. The supernatants were obtained by centrifugation (10,000 rpm, 20 min, 4 °C) and diluted to ethanol (1:1). EB content was measured fluorescent at 600 nm wavelengths with a spectrophotometer. The amount of EB present in the tissue samples was quantified using a linear regression standard curve derived from eleven concentrations (0–10^4^ ng/ml) of the dye and expressed as µg/g of tissue.

In addition, after transcardinally perfusion, brains were removed after 15-min perfusion fixation at 4 °C and then immersed in the same fixative solution overnight at 4 °C. Next, the brains were immersed in 25% sucrose in 0.1 M PB for 24 h and frozen in liquid nitrogen. Coronal sections were cut on a cryostat at −20 °C and stored at −80 °C until use, the size is 15 µm. The sections were washed with 0.01 M PBS, and then the sections were observed using BIOREVO BZ-X710 (Keyence, Osaka, Japan) at 600 nm wavelengths.

### Neurological deficit evaluation

Garcia test and grid walking test were conducted at 1 or 3 days after surgery, as described previously^29^. The Garcia scores (3 to 18) were calculated by combining the scores of the following 6 tests: spontaneous activity, symmetry in the movement of the 4 limbs, forepaw outstretching, climbing, body proprioception, and response to vibrissae touch. Each test sore had a total of 3 possible points according to the neurological presentation. In addition, to evaluate the mice forelimb motor function, the grid walking test was performed. The number of step errors was counted for 2 min during spontaneous movement on a grid, which is 0.24-mm wide and 10-mm^2^ opening.

### Neuronal damage area evaluation

Brain frozen slices were cut as described above. The slices were stained with Cresyl Violet to estimate the lesion volume as described previously^34^. Brain slices were observed under a BIOREVO BZ-X710 and lesional volume was analyzed with Image-J software version 1.43 h by measuring band intensities.

### *In vivo* western blotting analysis

Mice were deeply anesthetized and decapitated at 3 days after surgery. These brains were immediately removed, divided into hemisphere, and harvested peri-damaged region in striatum. Tissues were homogenized in 10 ml/g tissue ice-cold lysis buffer. A 10-µg protein sample was subjected to electrophoresis, following experimental procedure was described above. The following primary antibodies were used: HO-1 (1:200; Santa Cruz), platelet-derived growth factor receptor-β (PDGFR-β) (1:200; Santa Cruz), VE-cadherin (1:1000; Abcam), occludin (1:2000; Abcam), β-actin (1:2000; Sigma-Aldrich). Used antibodies were summarized in Table 1.

Immunoreactive bands were visualized using ImmunoStar LD. Band intensities were measured using LAS-4000. Expression level differences were analyzed with Image-J software by measuring band intensities^29,30^.

### Iron accumulation *in vivo* brain slices by using Si RhoNox-1 and antibodies

Brain slices were washed with PBS, and incubated with Si-RhoNox-1 (5 µM) for 1 h. After incubation, the slices were washed twice with PBS, and then the slices were observed under a confocal microscope and BIOREVO BZ-X710 at ex/em: 645/660 nm. (Vehicle, n = 3; Hemin, n = 3; Blood, n = 3).

In immunostaining assessment, the brain sections were washed with 0.01 M PBS, and preincubated with 5% goat serum (Vector Laboratories Inc., Burlingame, CA, USA) in 0.01 M PBS for 1 h at room temperature. Next, the sections were incubated overnight at 4 °C with the primary rabbit monoclonal antibody to Ferritin (1:100) in 5% goat serum. The following day, after washing for 5 min twice, the sections were incubated for 1 h with secondary antibody (Alexa Fluor 488 goat anti-rabbit, 1:1000; Thermo Fisher Scientific) under protection from light. After washings, the sections were preincubated with M.O.M. blocking reagent (M.O.M. immunodetection kit; Vector) in PBS for 1 h at room temperature. After washings, the sections were incubated overnight at 4 °C with the primary antibodies against PDGFR-β (1:100, Santa cruz) and CD31 (1:100, BD Biosciences) in M.O.M. diluents mixed with PBS (PBS: M.O.M. concentrate = 25:2). The next day, after washings, the sections were incubated for 30 min with the secondary antibody (Alexa Fluor 633 goat anti-mouse, 1:1000; Thermo Fisher Scientific) and Hoechist 33342 (1:1000) at room temperature. After washings, the sections were then observed under a confocal microscope. (Hemin, n = 3) Used antibodies were summarized in Table 1.

### The effect of iron chelator administration *in vivo* ICH model

Mice were randomly divided 4 groups; sham (only scalp incision), vehicle (saline injection into brain parenchyma and saline intraperitoneal administration, (i.p.), hemin (Hemin 10 mM/10 µL injection into brain parenchyma and saline administration, (i.p.), BP (Hemin 10 mM/10 µL injection into brain parenchymal tissue and BP administration, 40 mg/kg, i.p.). BP or saline administrations was at 2 h and 6 h after injection, and then once daily for 3 days. The BP dose was based a on previously report^49^. The final concentration of DMSO in BP or vehicle solution was 1%. In each group, body weight measurement and neurological deficit evaluation were performed.

### Statistic analysis

Data are presented as means ± standard error (SE) or standard deviation (SD) and were analyzed using the Statistical Package for the Social Science 15.0 J for Windows software (SPSS Japan Inc., Tokyo, Japan). Significant differences were determined by using Student’s or Welch’s *t*-test for two group comparisons, Mann Whitney *U*-test for nonparametric values and one-way analysis of variance (ANOVA) was followed Dunnett’ or Tukey’s test for multiple pair wise comparisons. In correlation test, we performed Spearman’s rank correlation coefficient. Results of p < 0.05 were considered.

## Supplementary information

Supplemental materials
