# Supplementary material for: Intracellular Fe2+ accumulation in endothelial cells and pericytes induces blood-brain barrier dysfunction in secondary brain injury after brain hemorrhage
Source: Sci Rep. 2019 Apr 17;9:6228. doi: 10.1038/s41598-019-42370-z (PMC6470176; doi:10.1038/s41598-019-42370-z)

For *Scientific Reports*

# Intracellular $Fe^{2+}$ accumulation in endothelial cells and pericytes induces blood-brain barrier dysfunction in secondary brain injury after brain hemorrhage

Takahiko Imai[1], Sena Iwata[1], Tasuku Hirayama[2], Hideko Nagasawa[2], Shinsuke Nakamura[1], Masamitsu Shimazawa[1], Hideaki Hara[1,*]

[1]Molecular Pharmacology, Department of Biofunctional Evaluation, Gifu Pharmaceutical University, Gifu 501-1196, Japan.

[2] Pharmaceutical and Medicinal Chemistry, Gifu Pharmaceutical University, Gifu 501-1196, Japan.

***Corresponding author:** Professor H Hara, PhD, RPh, Molecular Pharmacology, Department of Biofunctional Evaluation, Gifu Pharmaceutical University, 1-25-4 Daigaku-nishi, Gifu 501-1196, Japan. Tel and Fax: +81-58-230-8126, E-mail: hidehara@gifu-pu.ac.jp

**Supplemental materials**

**Results**

**Human Hb damaged BBB components via inducing ROS over-production and BP ameliorated Hb-induced harmful effects (Figure 1)**

Cell death rate after Hb treatment, **HBMVECs**, Control, 2.31 ± 0.32%; 1 μM, 4.28 ± 0.16%; 10 μM, 6.66 ± 0.66%; 25 μM, 7.90 ± 0.39%; **HBMVPs**, Control, 2.58 ± 0.31%; 1 μM, 8.04 ± 1.15%; 10 μM, 9.29 ± 1.55%; 25 μM, 12.8 ± 2.03% (Figure1B)

Cell death rate after Hb treatment with BP, **HBMVECs**, Control, 1.40 ± 0.08%; Hb,, 6.17 ± 0.79%; BP co-treatment, 2.08 ± 0.22%; BP only, 1.75 ± 0.37%; **HBMVPs**, Control, 2.68 ± 0.81%; Hb,, 10.01 ± 0.96%; BP co-treatment, 3.62 ± 0.69%; BP only, 2.47 ± 0.97% (Figure 1D).

ROS production rate compared to Control after Hb treatment with BP, **HBMVECs**, Hb, 688.07 ± 21.09%; BP co-treatment, 371.41 ± 6.56%; BP only, 59.99 ± 3.38%; **HBMVPs**, Hb, 980.61 ± 166.18%; BP co-treatment, 608.32 ± 31.77%; BP only, 163.12 ± 9.11% (Figure 1E).

HO-1 expression compared to Control after Hb treatment, **HBMVECs**, 1 μM, 1.18 ± 0.12%; 10 μM, 2.31 ± 0.28%; **HBMVPs**, 1 μM, 2.64 ± 0.38%; 10 μM, 4.60 ± 0.54% (Figure 1F).

**$Fe^{2+}$ regent induced intracellular $Fe^{2+}$ accumulation and cell death in both endothelial cells and pericytes (Figure 2)**

The intracellular $Fe^{2+}$ accumulation ($Fe^{2+}_{intra}$) level compared to Control after FAS treatment, **HBMVECs**, Control, 100 ± 7.25%; 30 μM, 57.72 ± 4.81%; 100 μM, 93.43 ± 20.36%; 300 μM, 239.69 ± 61.02%; **HBMVPs,** Control, 100 ± 13.28%; 30 μM, 128.87 ± 7.62%; 100 μM, 169.87 ± 9.96%; 300 μM, 187.45 ± 16.71% (Figure 2B).

The intracellular $Fe^{2+}$ accumulation ($Fe^{2+}_{intra}$) level compared to Control after FAS treatment with BP, **HBMVECs**, Control, 100 ± 8.99%; FAS, 207.71 ± 17.00%; BP co-treatment, 51.42 ± 0.60%; BP only, 54.63±7.56%; **HBMVPs**, Control, 100 ± 5.85%; FAS, 198.46 ± 9.99%; BP co-treatment, 34.45 ± 3.36%; BP only, 49.38 ± 1.56% (Figure 2C).

Cell death rate after FAS treatment for 24 h, **HBMVECs**, Control, 0.95 ± 0.11%; 30 μM, 2.41 ± 0.54%; 100 μM, 3.42 ± 0.44%; 300 μM, 4.52 ± 0.38%; **HBMVPs**, Control, 0.52 ± 0.11%; 30 μM, 0.89 ± 0.36%; 100 μM, 2.18 ± 0.32%; 300 μM, 0.70 ± 0.21% (Figure 2D).

**Intracellular $Fe^{2+}$ accumulation was correlated to cell death in both endothelial**

**cells and pericytes (Figure 3)**

Cell death rate after FAS treatment for 0.5, 2, 4, 6, 12 and 24 h, **HBMVECs**, 0.5 h, Control, 1.19 ± 0.39%; 30 µM, 0.87 ± 0.11%; 100 µM, 1.76 ± 0.30%; 300 µM, 1.80 ± 0.42%; 2 h, Control, 0.93 ± 0.35%; 30 µM, 0.89 ± 0.37%; 100 µM, 0.46 ± 0.09%; 300 µM, 1.48 ± 0.41%; 4 h, Control, 0.45 ± 0.17%; 30 µM, 0.74 ± 0.29%; 100 µM, 0.70 ± 0.15%; 300 µM, 1.10 ± 0.16%; 6 h, Control, 0.66 ± 0.32%; 30 µM, 0.49 ± 0.17%; 100 µM, 0.96 ± 0.38%; 300 µM, 0.86 ± 0.25%; 12 h, Control, 0.30 ± 0.03%; 30 µM, 0.68 ± 0.10%; 100 µM, 0.59 ± 0.11%; 300 µM, 0.72 ± 0.13%; 24 h, Control, 0.78 ± 0.08%; 30 µM, 1.87 ± 0.14%; 100 µM, 3.52 ± 0.39%; 300 µM, 4.24 ± 0.30%; **HBMVPs**, 0.5 h, Control, 1.12 ± 0.21%; 30 µM, 1.20 ± 0.23%; 100 µM, 1.21 ± 0.13%; 300 µM, 0.99 ± 0.13%; 2 h, Control, 0.57 ± 0.09%; 30 µM, 0.84 ± 0.23%; 100 µM, 0.95 ± 0.09%; 300 µM, 1.06 ± 0.14%; 4 h, Control, 0.94 ± 0.21%; 30 µM, 1.47 ± 0.11%; 100 µM, 1.59 ± 0.12%; 300 µM, 1.49 ± 0.12%; 6 h, Control, 0.31 ± 0.05%; 30 µM, 1.78 ± 0.17%; 100 µM, 1.79 ± 0.09%; 300 µM, 1.97 ± 0.19%; 12 h, Control, 0.37 ± 0.08%; 30 µM, 2.07 ± 0.25%; 100 µM, 1.79 ± 0.10%; 300 µM, 2.66 ± 0.26%; 24 h, Control, 0.31 ± 0.04%; 30 µM, 1.94 ± 0.28%; 100 µM, 1.78 ± 0.16%; 300 µM, 1.80 ± 0.17%; (Figure 3B).

$Fe^{2+}_{intra}$ levels compared to Control after FAS treatment, **HBMVECs**, 0.5 h, Control, 100 ± 3.91%; 30 µM, 124.15 ± 7.97%; 100 µM, 142.37 ± 20.28%; 300 µM, 219.59 ±

12.48%; 6 h, Control, 100 ± 9.84%; 30 µM, 122.19 ± 10.43%; 100 µM, 236.33 ± 19.37%; 300 µM, 281.94 ± 13.52%; 24 h, Control, 100 ± 6.10%; 30 µM, 119.76 ± 5.06%; 100 µM, 469.04 ± 11.12%; 300 µM, 482.44 ± 10.81%; **HBMVPs**, 0.5 h, Control, 100 ± 16.29%; 30 µM, 199.40 ± 10.25%; 100 µM, 220.14 ± 12.75%; 300 µM, 230.03 ± 15.23%; 6 h, Control, 100 ± 3.16%; 30 µM, 154.43 ± 23.40%; 100 µM, 195.24 ± 18.87%; 300 µM, 247.55 ± 26.85%; 24 h, Control, 100 ± 5.81%; 30 µM, 324.76 ± 56.56%; 100 µM, 356.68 ± 19.29%; 300 µM, 452.23 ± 18.57% (Figure 3C).

The correlation between $Fe^{2+}_{intra}$ levels and cell death, total, **HBMVECs**, y = 0.004839x+0.631322, r = 0.283941; **HBMVPs**, y = 0.002938x+0.575016, r = 0.570069 (Figure 3D), 0.5 h, **HBMVECs**, r = 0.372174; **HBMVPs**, r = 0.061739 (Supplemental Figure 1A), 6 h, **HBMVECs**, r = 0.272174; **HBMVPs**, r = 0.562609 (Supplemental Figure 1B), 24 h, **HBMVECs**, r = 0.829565; **HBMVPs**, r = 0.515652 (Supplemental Figure 1C).

**Hb or hemin induced $Fe^{2+}$ accumulation in both endothelial cells and pericytes (Figure 4)**

$Fe^{2+}_{intra}$ level compared to Control after Hb treatment, **HBMVECs**, Control, 100 ±

4.55%; 1 µM, 294.79 ± 5.87%; 10 µM, 601.96 ± 34.7%; 25 µM, 779.29 ± 71.37%; **HBMVPs**, Control, 100 ± 17.17%; 1 µM, 173.83 ± 14.00%; 10 µM, 354.64 ± 28.85%; 25 µM, 455.48 ± 24.97% (Figure 4B).

$Fe^{2+}_{intra}$ level compared to Control after Hb treatment with BP, **HBMVECs**, Control, 100 ± 5.91%; Hb, 541.02 ± 28.62%; BP co-treatment, 420.10 ± 20.94%; BP only, 59.17 ± 2.94%; **HBMVPs**, Control, 100 ± 11.27%; Hb, 321.58±18.97%; BP co-treatment, 220.93 ± 31.68%; BP only, 22.38 ± 1.20% (Figure 4C).

$Fe^{2+}_{intra}$ level compared to Control after hemin treatment, **HBMVECs**, Control, 100 ± 8.74%; 1 µM, 305.70 ± 12.54%; 10 µM, 349.71 ± 17.80%; 50 µM, 398.40 ± 13.01%; **HBMVPs**, Control, 100 ± 6.38%; 1 µM, 214.82 ± 7.78%; 10 µM, 345.71 ± 12.72%; 50 µM, 394.05 ± 3.54% (Figure 4D).

$Fe^{2+}_{intra}$ level compared to Control after he treatment with BP, **HBMVECs**, Control, 100 ± 17.23%; Hemin, 854.57 ± 53.87%; BP co-treatment, 687.46 ± 32.22%; BP only, 74.68 ± 4.72%; **HBMVPs**, Control, 100 ± 10.58%; Hemin, 888.94 ± 24.04%; BP co-treatment, 809.25 ± 15.11%; BP only, 58.15 ± 4.27% (Figure 4E).

**Hemin induced the harmful effects on BBB composed cells and induced barrier dysfunction via iron-mediated apoptosis**

Cell viability compared to Control after hemin treatment, **HBMVECs**, Control, 100 ± 3.44%; 1 μM, 92.68 ± 3.18%; 10 μM, 86.87 ± 1.46%; 50 μM, 10.57 ± 0.87%; **HBMVPs**, Control, 100 ± 1.09%; 1 μM, 91.13 ± 2.30%; 10 μM, 82.80 ± 1.76%; 50 μM, 25.17 ± 0.68% (Figure 5B).

Cell death rate after hemin treatment with BP, **HBMVECs**, Control, 1.21 ± 0.27%; Hemin, 88.49 ± 3.32%; BP co-treatment, 9.41 ± 2.02%; BP only, 1.69 ± 0.75%; **HBMVPs**, Control, 0.90 ± 0.48%; Hemin, 59.44 ± 4.63%; BP co-treatment, 13.44 ± 5.77%; BP only, 0.61 ± 0.13% (Figure 4F).

ROS production rate compared to Control after hemin treatment with BP, Control, 100 ± 31.37%; Hemin, 597.29 ± 57.64%; BP co-treatment, 207.03 ± 21.00%; BP only, 75.91 ± 8.26%; **HBMVPs**, Control, 100 ± 6.92%; Hemin, 817.17 ± 48.23%; BP co-treatment, 345.75 ± 23.00%; BP only, 113.17 ± 7.07% (Figure 5D).

TEER value compared to Control after Hemin treatment, Control, 100 ± 12.65%; Hemin, 45.33 ± 10.14% (Figure 5F)

FITC-dextran permeability rate compared to Control after Hemin treatment, Control, 100 ± 39.39%; Hemin, 263.18 ± 41.30% (Figure 5G)

TEER value compared to Control after Hb treatment, Control, 100 ± 7.47%; Hb, 19.80 ± 4.77%.

FITC-dextran permeability rate compared to Control after Hemin treatment, Control, 100 ± 10.02%; Hb, 141.10 ± 14.15% (Supplemental Figure 2B)

**Hemin injection induced brain damage in an *in vivo* mice model**

EB dye leakage weight in brain tissue after hemin injection, striatum region (Ipsi), Sham, 1.80 ± 0.65 µg/g; 1 day, 29.74 ± 6.38 µg/g; 3 day, 37.21 ± 7.93 µg/g; other region (Ipsi), Sham, 1.91 ± 0.79 µg/g; 1 day, 3.51 ± 0.94 µg/g; 3 day, 5.75 ± 0.64 µg/g; striatum region (Contra), Sham, 3.21 ± 1.37 µg/g; 1 day, 10.49 ± 2.20 µg/g; 3 day, 8.83 ± 1.58 µg/g; other region (Contra), Sham, 1.21 ± 0.78 µg/g; 1 day, 2.20 ± 0.52 µg/g; 3 day, 4.50 ± 0.99 µg/g (Figure 6C).

Neurological deficits score after hemin injection, Garcia test, Sham, 18 ± 0; saline 1 day, 16.80 ± 0.50; hemin 1 day, 15.44 ± 0.67; saline 3 day, 17.6 ± 0.24; hemin 3 day, 16.33 ± 0.33, Grid walking test, Sham, 1.86 ± 0.35; saline 1 day, 5.80 ± 0.80; hemin 1 day, 10.67 ± 1.44; saline 3 day, 3.4 ± 0.75; hemin 3 day, 11.0 ± 1.86 (Figure 6D).

Neuronal damage area compared to Vehicle, Hemin, 4.94±0.59% (Figure 6E).

**Hemin injection altered BBB integrity and accumulated iron in both endothelial cells and pericytes**

Proteins expression compared to Vehicle after hemin injection, HO-1, 3.13 ± 0.40%; VE-cadherin, 0.22 ± 0.07%; Occludin, 0.58 ± 0.06%; PDGFR-β, 0.32 ± 0.13% (Figure 7A).

Proteins expression compared to Control after Hb or hemin treatment, cleaved caspase-3, Hb, 4.78 ± 0.50%; Hemin, 11.83 ± 0.74%; HO-1, Hb, 2.38 ± 0.20%; Hemin, 3.80 ± 0.8%; Ferritin, Hb, 6.09 ± 1.05%; Hemin, 21.40 ± 3.95% (Supplemental Figure 3B).

**An iron chelator ameliorates neurological dysfunction in the murine hemin injection ICH model**

Neurological deficits score after hemin injection, Sham, 7.67 ± 3.18; Vehicle, 10.22 ± 1.33; Hemin, 18.78 ± 2.34; BP, 12.67 ± 1.36 (Figure 8B).

**Supplemental Figure 1. The correlation between cell death and $Fe^{2+}_{intra}$ levels after FAS, a $Fe^{2+}$ reagent in both endothelial cells and pericytes.**

(A) The correlation analysis at 0.5 h after FAS treatment. (B) The correlation analysis at 6 h after FAS treatment. (C) The data was analyzed with Spearman's rank correlation coefficient (n = 6).

**Supplemental Figure 2. Hb induced endothelial barrier dysfunction.**

(A) Experimental protocol of TEER value measurement and FITC-dextran permeability assay after Hb treatment (10 μM). (B) The obtained TEER values (n = 4). (C) Permeability rate (n = 4). $^{**}p < 0.01$, $^{*}p < 0.05$ vs. Control. The data was analyzed with the Student’s *t*-test. The data are expressed as the mean ± SE.

**Supplemental Figure 3. Hb or hemin altered the expression of several proteins in endothelial cells.**

(A) Experimental protocol of western blotting analysis after Hb (10 μM) or hemin (50 μM) treatment (n = 5). (B) The expression levels of several proteins. The upper images are representative bands and the lower graphs comprise the quantitative data. $^{**}p < 0.01$, $^{*}p < 0.05$ vs. Control. The data was analyzed with the Student’s *t* test or

Welch's *t* test. The data are expressed as the mean ± SE.

**Supplemental Figure 4. Full bands in Figure 1F.**

**Supplemental Figure 5. Full bands in Figure 5I.**

**Supplemental Figure 6. Full bands in Figure 7A.**

**Supplemental Figure 7. Full bands in Supplemental Figure 3.**

# Supplemental Figure 1

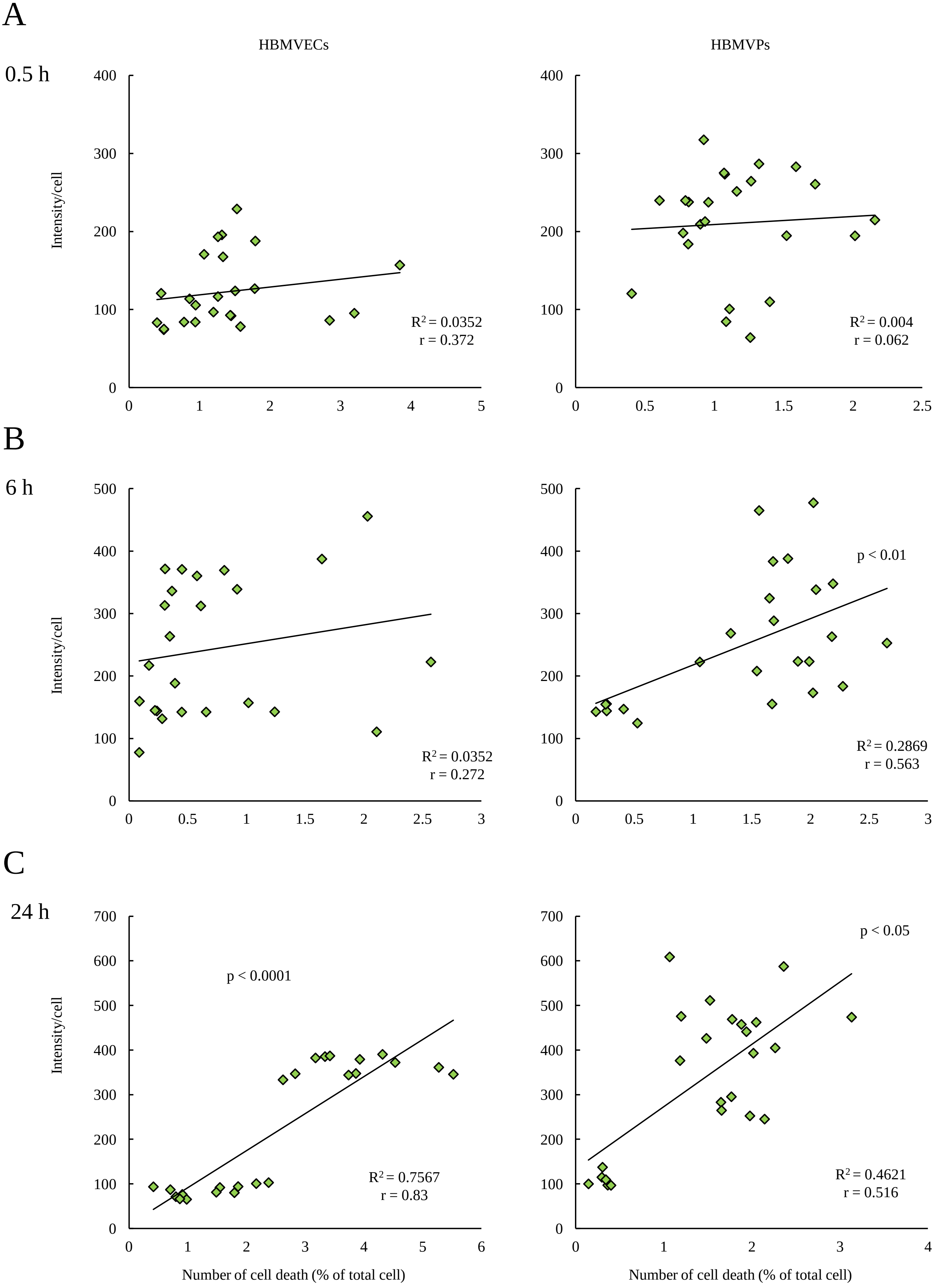

# Supplemental Figure 2

A

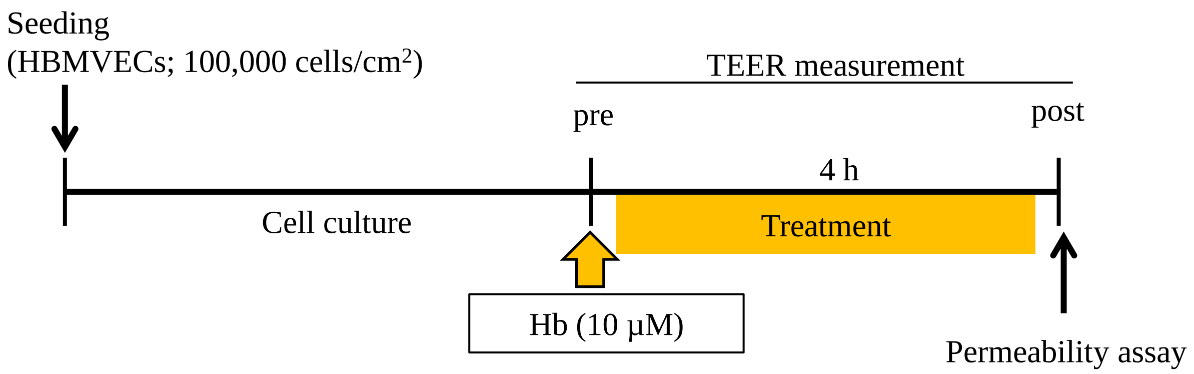


B

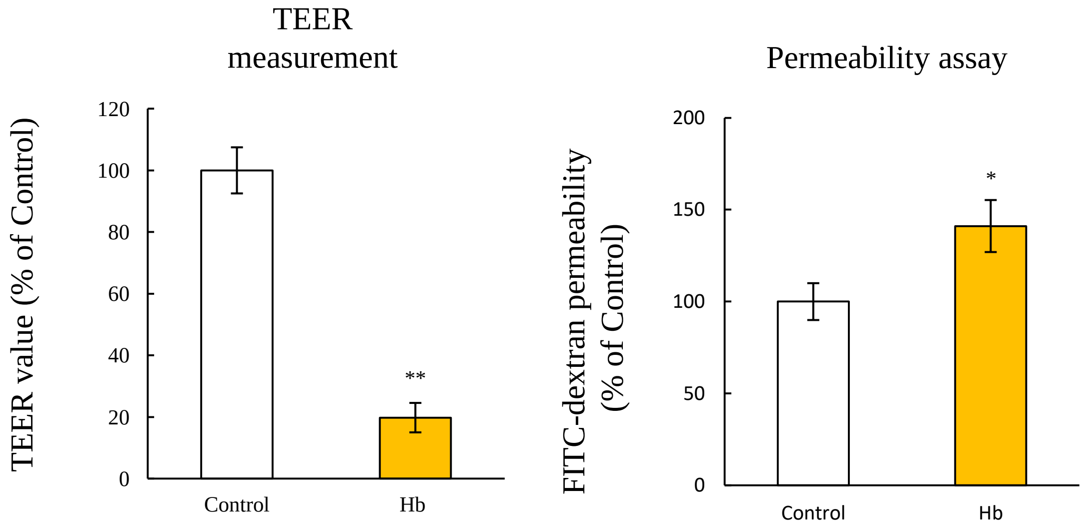

# Supplemental Figure 3

A

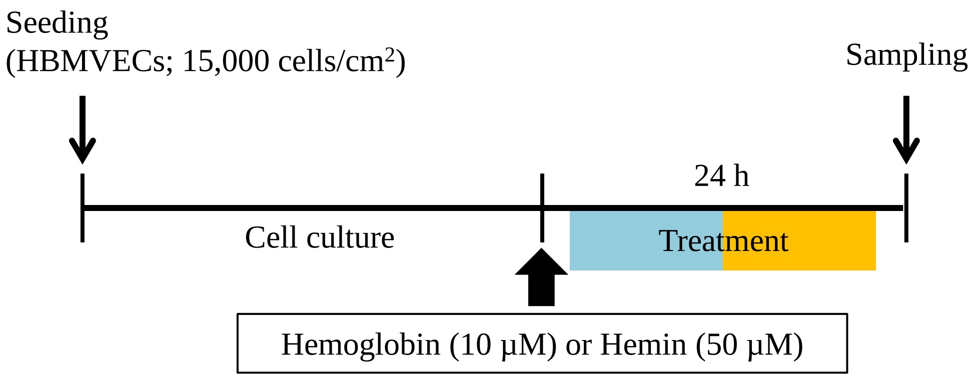


B

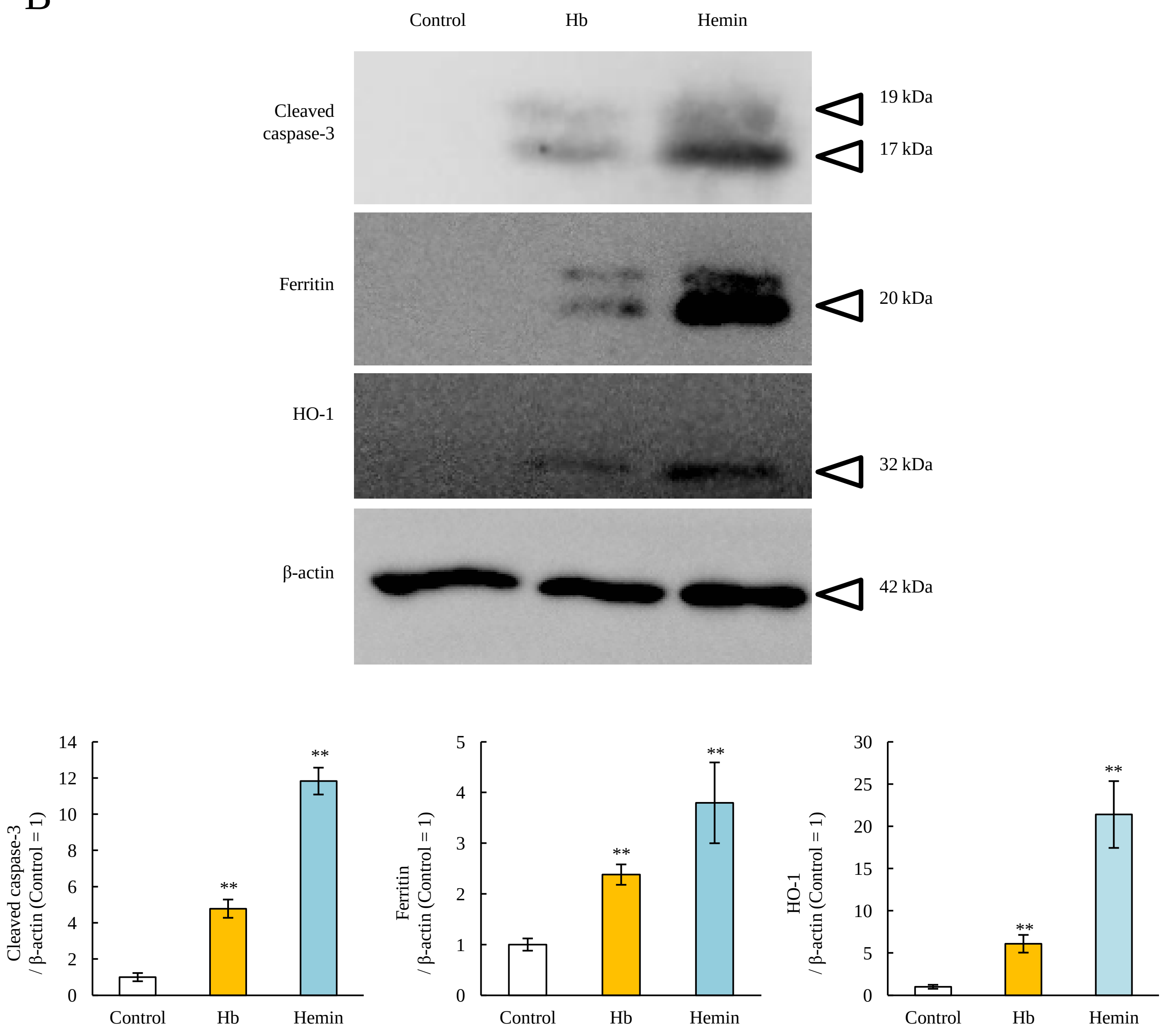

Supplemental Figure 4

# Full bands in Figure 1F

HBMVECs

| | Hb | | | Hb | | | Hb | | | Hb | |
|---|---|---|---|---|---|---|---|---|---|---|---|
| C | 1 | 10 | C | 1 | 10 | C | 1 | 10 | C | 1 | 10 |

**HO-1**

32 kDa

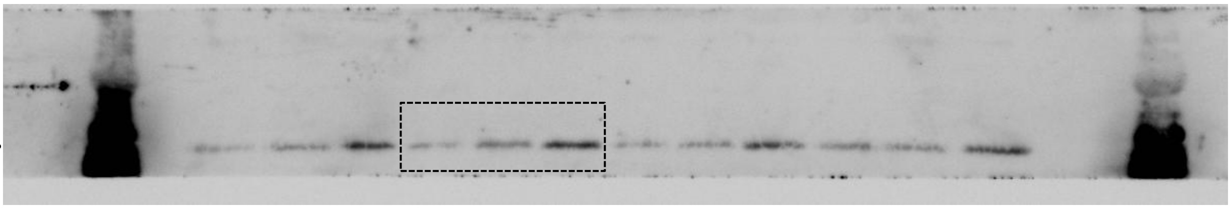

**β-actin**

42 kDa

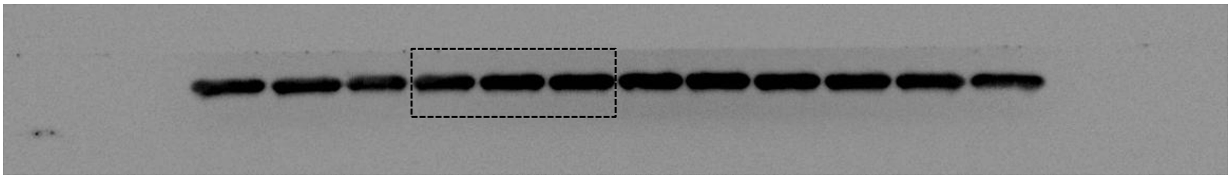

HBMVPs

**HO-1**

32 kDa

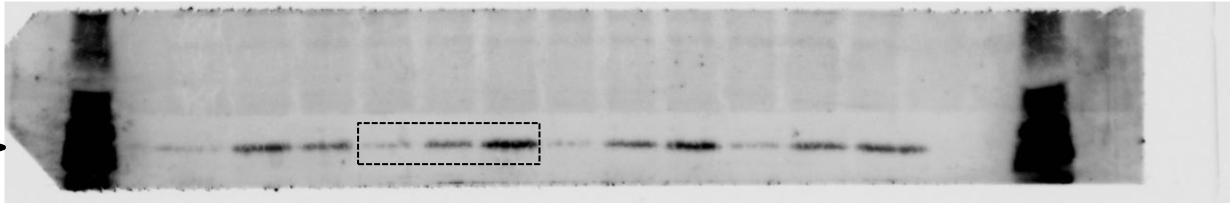

**β-actin**

42 kDa

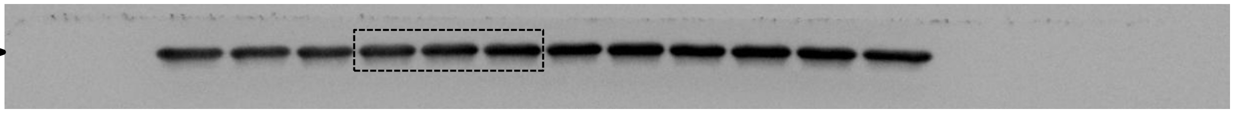

# Full bands in Figure 5I

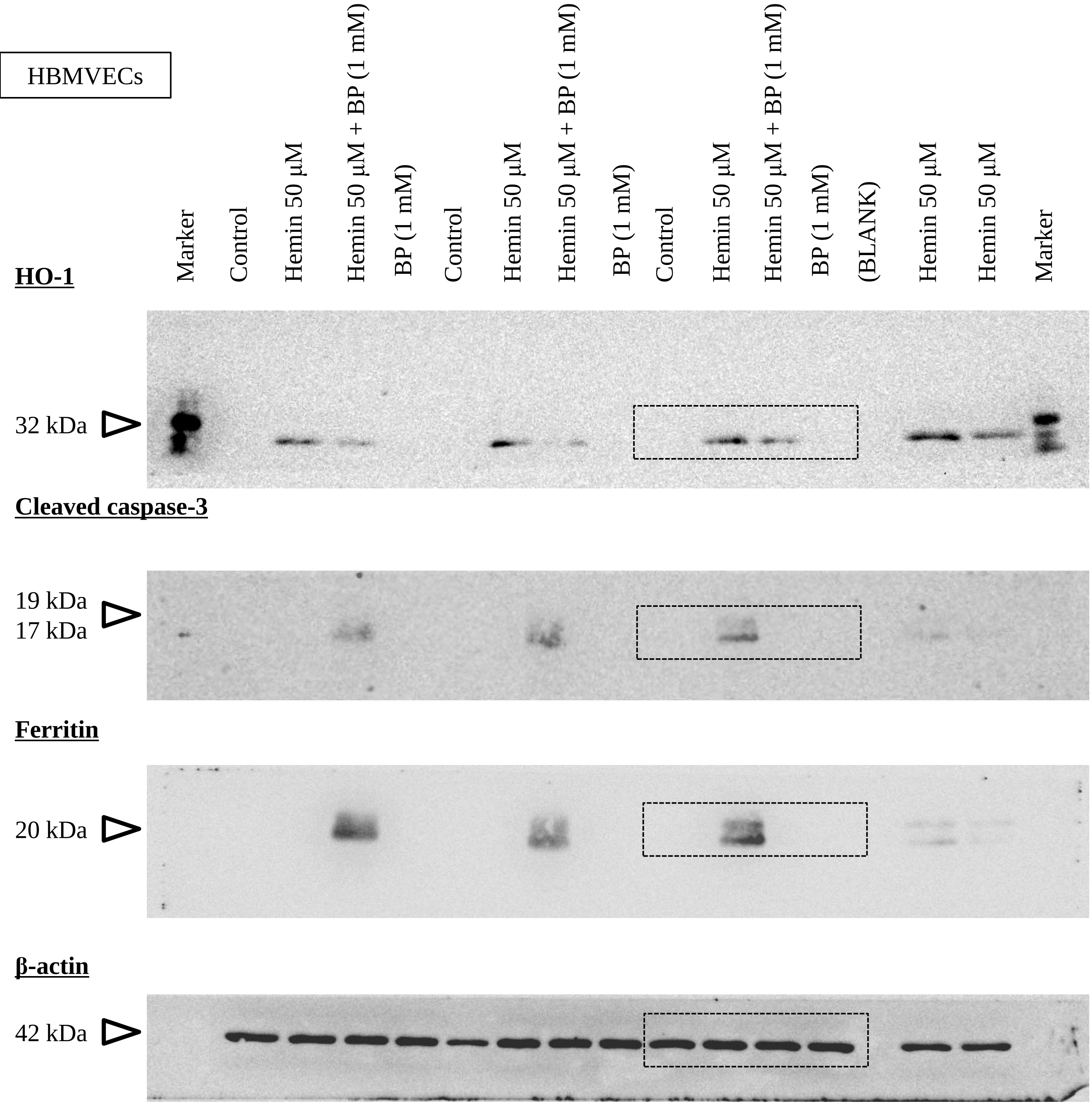

Supplemental Figure 6

# Full bands in Figure 7A

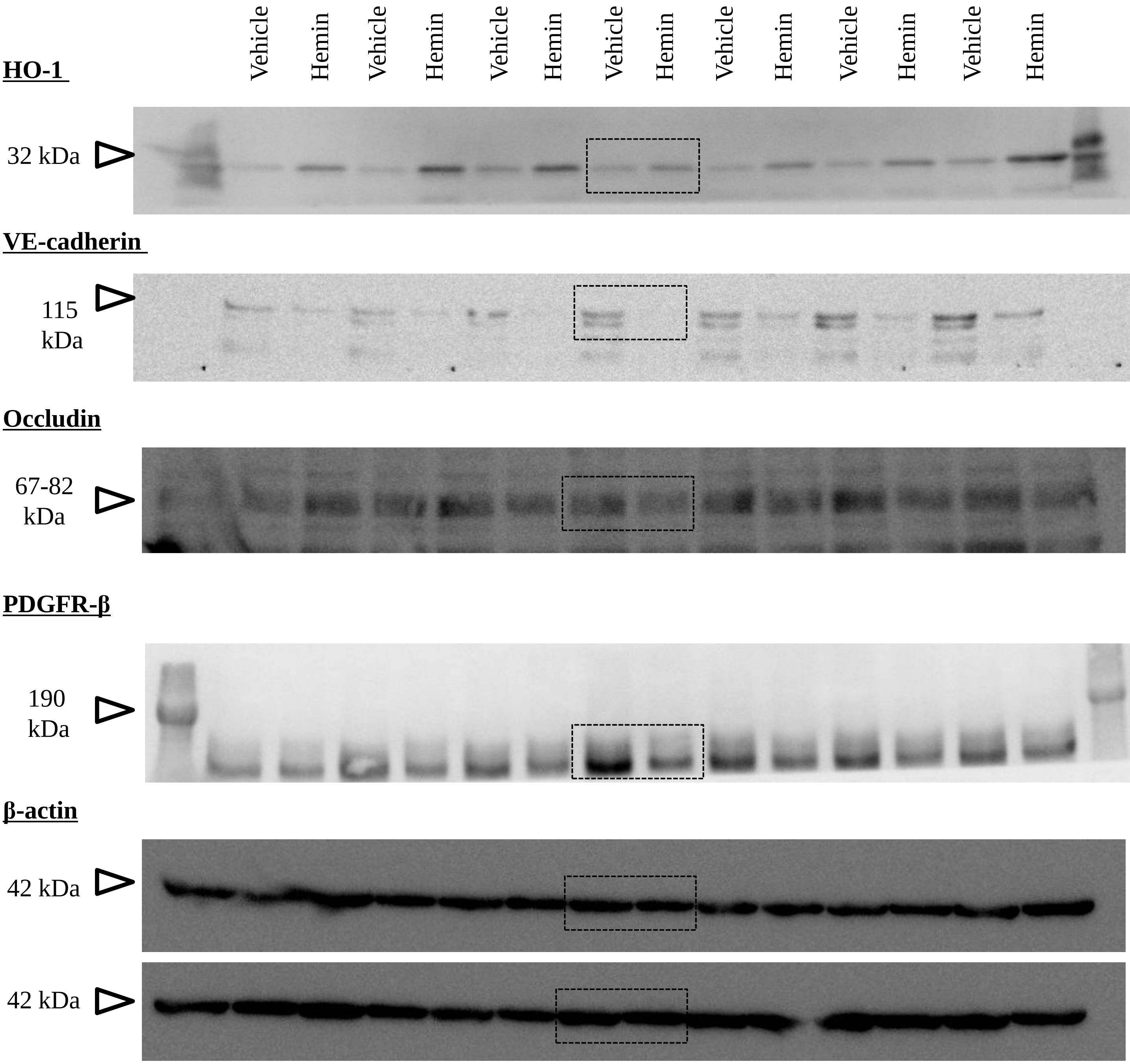

Supplemental Figure 7

# Full bands in Supplemental Figure 3

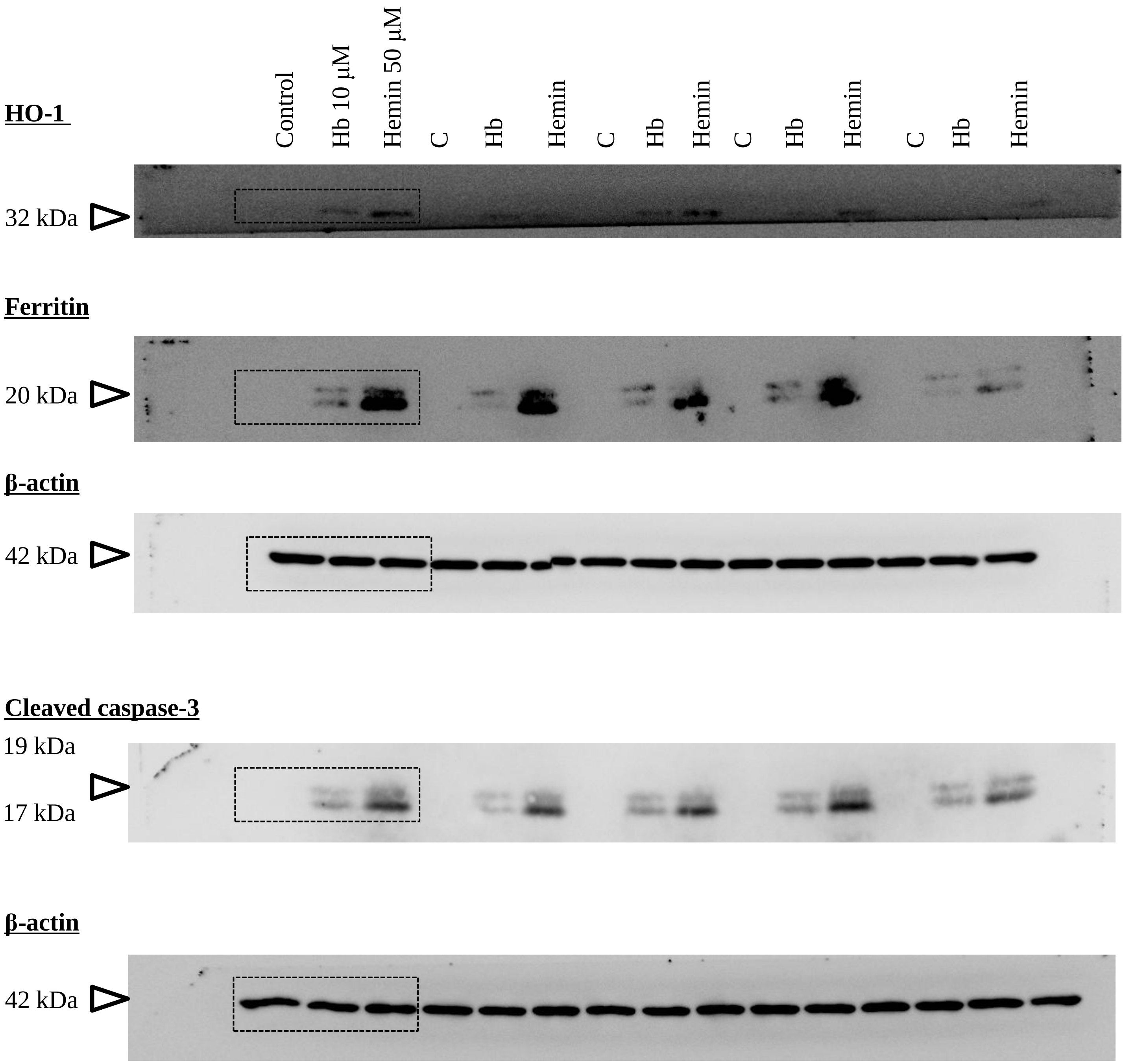

Supplement: Supplementary file 1 — Supplemental materials [file 41598_2019_42370_MOESM1_ESM.pdf]
